# Implantation Serine Proteinase 1 Exhibits Mixed Substrate Specificity that Silences Signaling via Proteinase-Activated Receptors

**DOI:** 10.1371/journal.pone.0027888

**Published:** 2011-11-23

**Authors:** Navneet Sharma, Rajeev Kumar, Bernard Renaux, Mahmoud Saifeddine, Sandra Nishikawa, Koichiro Mihara, Rithwik Ramachandran, Morley D. Hollenberg, Derrick E. Rancourt

**Affiliations:** 1 Department of Biochemistry and Molecular Biology, University of Calgary, Calgary, Canada; 2 Department of Physiology and Pharmacology University of Calgary, Calgary, Canada; 3 Department of Medicine, Faculty of Medicine, University of Calgary, Calgary, Canada; State Key Laboratory of Reproductive Biology, Institute of Zoology, Chinese Academy of Sciences, China

## Abstract

Implantation S1 family serine proteinases (ISPs) are tryptases involved in embryo hatching and uterine implantation in the mouse. The two different ISP proteins (ISP1 and ISP2) have been detected in both pre- and post-implantation embryo tissue. To date, native ISP obtained from uterus and blastocyst tissues has been isolated only as an active hetero-dimer that exhibits trypsin-like substrate specificity. We hypothesised that in isolation, ISP1 might have a unique substrate specificity that could relate to its role when expressed alone in individual tissues. Thus, we isolated recombinant ISP1 expressed in *Pichia pastoris* and evaluated its substrate specificity. Using several chromogenic substrates and serine proteinase inhibitors, we demonstrate that ISP1 exhibits trypsin-like substrate specificity, having a preference for lysine over arginine at the P1 position. Phage display peptide mimetics revealed an expanded but mixed substrate specificity of ISP1, including chymotryptic and elastase activity. Based upon targets observed using phage display, we hypothesised that ISP1 might signal to cells by cleaving and activating proteinase-activated receptors (PARs) and therefore assessed PARs 1, 2 and 4 as potential ISP1 targets. We observed that ISP1 silenced enzyme-triggered PAR signaling by receptor-disarming. This PAR-disarming action of ISP1 may be important for embryo development and implantation.

## Introduction

The implantation serine proteinases, ISP1 & 2, are two related S1-family serine proteinases that are tandemly localized in a cluster of tryptase genes found on mouse chromosome 17A3.3 [1]. Unlike many of the other tryptases, which are found primarily in mast cells, the ISPs are expressed in the embryo and the uterine decidua during the time of embryo implantation [2]. The first ISP gene to be characterized (ISP1) was initially detected in the pre-implantation embryo [3]. Anti-sense RNA disruption of ISP1 gene expression prevented embryo hatching and outgrowth *in vitro* [3]. Subsequently, ISP1 and ISP2 gene expression was detected in the uterine endometrial glands during the ‘*window of implantation’* [4], [5]. Artificial pregnancy experiments demonstrate that both ISP genes are up-regulated by progesterone [4], [5]. Both ISPs are secreted from the endometrial glands into uterine fluid on day 4 of pregnancy, just prior to the commencement of implantation [6]. This appearance of enzyme in the glands and uterine fluid is negatively regulated by estrogen, such that both ISP proteins appear in the uterine fluid shortly after the estrogen spike synchronizes uterine-embryo receptivity to enable the commencement of implantation [6]. Interestingly, ISP2 antibodies have been found to abrogate murine embryo implantation, verifying an important role for ISPs [7]. Synthetic inhibitors of ISP activity have also demonstrated a potential role for ISPs in the early stages of murine embryo invasion *in vitro* and implantation *in vivo* [2], [8]. ISP protein and proteolytic activity are found in uterine fluid at sites localized to embryo invasion [1], [3]. The cognate tryptase is minimally a hetero-dimer comprised of both ISP1 and ISP2 enzymes, although homo-dimers have not been ruled out [2].

Preliminary studies using chromogenic p-nitroanilide conjugated synthetic peptide substrates have indicated that the ISP1/ISP2 hetero-dimer has ‘trypsin-like’ specificity [2]. Recent studies employing a bacteriophage display of small peptides have not only confirmed this substrate specificity but have also demonstrated a preference for non-polar amino acid residues at the P1′ as well as at the P2 positions [9]. Since these studies were performed using purified native ISP1/ISP2 enzyme complex [2], we have been interested in expressing recombinant ISPs in order to investigate the activity of each individual enzyme and to pursue enzyme structure-activity studies.

A number of different high throughput screening methods including libraries of chromogenic or fluorogenic substrates (in solution and/or on chip), have been devised to determine the substrate specificity of proteinases. Phage display has been developed as a more efficient and unbiased approach. So far, the phage display technology has been employed for displaying peptides/proteins for many different applications, including: (a) generating highly specific antibodies, (b) studying protein-protein interaction and (c) evaluating the substrate specificity of proteinases [10], [11], [12]. The T7 bacteriophage display system has been employed successfully for determining the substrate specificities of rat mast cell proteinases 4 and 5 [13], [14], the native implantation serine proteinase ISP1/ISP2 hetero-dimeric complex and human kallikrein 6 [9].

Many physiological responses mediated by serine proteinases can occur by cleaving and activating the PAR family of G-protein coupled receptors (GPCRs) [15]–[21]. The four members of the PAR family, PARs 1 to 4, have a unique mechanism of activation that distinguishes them from other seven transmembrane GPCRs. PARs carry their own activating molecule in a masked state and receptor activation is achieved through proteolytic cleavage at a specific arginine site within the receptor N-terminus to reveal a cryptic tethered ligand that activates the receptor [15], [17], [18], [21]. In addition, PARs, with the exception of PAR_3_, are also activated by short synthetic peptides (PAR-activating peptides, or PAR-APs) derived from the sequences of the proteolytically revealed tethered ligand [19], [20]. In addition to the cleavage/activation of PARs, proteinases can also negatively regulate signaling via the PARs by ‘disarming’ the receptor through cleavage at a downstream non-receptor activating site to remove the tethered ligand. These truncated receptors, refractory to activation by their target enzymes (e.g. trypsin for PAR_2_) are therefore unable to signal in a physiological setting; but nonetheless remain responsive to PAR-APs [21].

In this study, we have used the methylotrophic yeast *Pichia pastoris* in order to express recombinant ISP1, also known as Mouse Prss28. Our aim was to evaluate the substrate specificity of this enzyme acting on its own, in the absence of ISP2. Our data demonstrate that recombinant ISP1 can exist in a monomeric form. To evaluate the substrate preference of monomeric ISP1, we studied: (a) the kinetics of cleavage of several small chromogenic synthetic peptide substrates, (b) the effects of serine proteinase inhibitors on this activity, (c) cleavage of a random hexameric library of phage displayed peptides and (d) cleavage of synthetic peptides with sequences based on the results obtained from the phage display approach. Finally, in view of the tryptic activity of ISP, we hypothesised that ISP1 could regulate PAR activity. Thus, we also assessed the ability of the enzyme: (a) to regulate the activity of PARs 1, 2 and 4 and (b) to cleave peptide sequences derived from the cleavage-activation domain and from extracellular loop-2 (ECL2) of PAR_2_, as we had done previously for trypsin IV [22]. Our data indicate that the ISP1 monomer has mixed substrate specificity with tryptic, chymotryptic and elastase characteristics and that ISP1 can target the PARs primarily by disarming them. These actions of ISP1 may enable it to play a physiological role in murine development or embryo implantation.

## Results

### Expression and Purification of recombinant ISP1

Although the full length cDNA sequence of ISP1 suggests that it is secreted as a pro-enzyme, we have previously only detected its mature enzymatically active form as a complex with ISP2 (9), when isolated from uterine fluid. Based upon this previous observation, we sought to express the enzymatically active mature form of ISP1 in the Pichia expression system using a protease deficient strain of *Pichia pastoris*. As mentioned, the designed cDNA was designed to generate the active enzyme, with an N-terminal sequence (IVGG) found in active tryptases [4]. Accordingly we introduced an in-frame fusion of mature protein cDNA sequence, so as to place the mature protein downstream of the *Pichia* signal peptide sequence in the vector PICZαB.

Recombinant ISP1 expression was seen after approximately 50 hours of fermentation and peaked at approximately 100 hours (Figure S1C). The growth profile of the organism was also demonstrated by measuring packed cell volume (Figure S1A). A steady rise in growth was observed after 36 hours of fermentation until the end of the run. No difference in the fermentation parameters and expression profile was observed in the transition from 1.0 L to 10.0 L scale-up. Therefore these parameters may be considered as optimum for the expression of rISP1 in *Pichia pastoris*.

**Figure 1:**
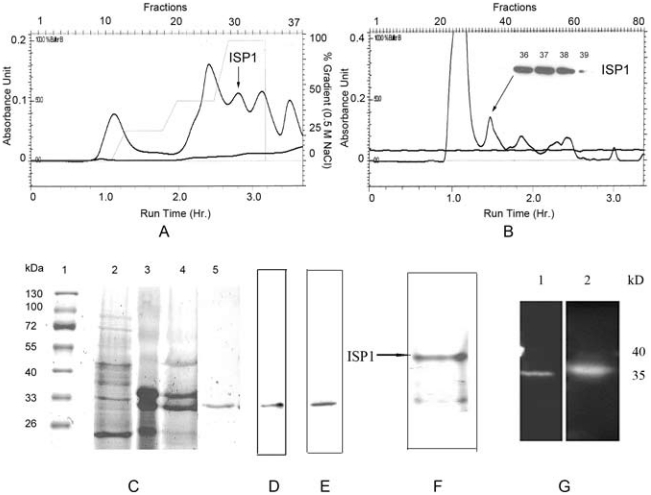
Purification of recombinant ISP1. (A) Ion exchange (DEAE-Sepharose) chromatography of harvested fermentation broth supernatant showing elution of rISP1 by a step gradient ranging from 0–0.5 M Sodium Chloride, (B) Gel filtration chromatography of the ion exchange-purified fractions of rISP1 (the arrow points towards the ISP1 fraction with the inset showing western blot results obtained using monoclonal anti-ISP1 antibodies), (C) Coomassie stained SDS-PAGE (10%) showing purification profile of fractions obtained at different steps of purification, lane 1 – Molecular weight marker (M/S Fermentas), lane 2 – fermentation broth supernatant (after removing cells), lane 3 – Eluted rISP1 fraction obtained after Ion exchange chromatography, lane 4 – Pooled fractions obtained after Superdex-200 Gel filtration chromatography step, lane 5 – Fraction obtained after purification with affinity chromatography (Benzamidine-Sepharose column), (D) Lane showing purified preparation of rISP1 obtained after 2^nd^ step of Gel Filtration Chromatography (Superdex-75), (E)Western blot analysis of purified rISP1 fraction using monoclonal anti-ISP1 antibodies, (F) Coomassie stained 8% native PAGE showing rISP1 as the major protein in the purified fraction after purification through ion exchange followed by gel filtration chromatography (G) Detection of ISP1 by activity based probe visualised by ECL plus, lane 1–20 mU Trypsin, lane 2–32 mU ISP1.

Figure 1 shows the FPLC chromatograms obtained upon purification of rISP1 with DEAE Sepharose (Figure 1A) and Superdex-75 (Figure 1B) columns. As seen in Figure 1A, ISP1 was eluted at the last step of the elution buffer gradient (100% Buffer B). A substantial amount of high molecular weight as well as low molecular weight non-ISP1 impurities were separated in the subsequent gel filtration step as shown in Figure 1B. Although only 3.9 fold purification occurred in the ion exchange chromatography step, a 100-fold purification was achieved in the gel filtration steps (Table 1 in Data S1). In total, a 340-fold purification was achieved by following this methodology. Hence, the specific activity of recombinant ISP1 increased from 0.54 units/mg protein to 180 units/mg. Figures 1C and 1D show the total protein profile of different fractions of ISP1, before and after purification, confirming the results shown in Table 1. The gels shown in Figure 1C (Lane 5) and 1D also demonstrate that the molecular weight of ISP1 monomer is ∼34 kDa. This size corresponded to the enzyme detected by western blot analysis (Figure 1E). Since the protein elutes at this size (∼34 kDa) upon gel filtration chromatography (calibration plot not shown), it appears that the recombinant ISP1 exists as a monomeric protein under the conditions of chromatography.

**Table 1:** Turnover rate (**kcat**), Michaelis Menton Constant (**Km**) and **kcat/Km** ratio of recombinant ISP1 for some synthetic chromogenic substrates.

	Substrate	TurnoverNo. (kcat)sec^−1^	MichaelisMentonConstant(Km) mM	kcat/Km (sec^−1^ mM^−1^)
1.	N-Benzoyl Argininep-nitroanilide (BAPNA)	2.2	0.61	3.6
2.	N-CBZ-Arginine-Arginine-Arginine-4 methoxynaphthylamide	0.7	0.099	7.1
3.	Isoleucine-Phenylalanine-Lysine p-nitroanilide	3.9	9.6	0.41
4.	Phenylalanine-Valine-Arginine p-nitroanilide	0.14	0.36	0.39
5.	Valine-Leucine-Lysinep-nitroanilide	Nil	Nil	Nil
6.	Tosyl-Glycine-Proline-Lysine p-nitroanilide	Nil	Nil	Nil
7.	Succinyl-Alanine-Alanine-Alanine p-nitro anilide	Nil	Nil	Nil
8.	Alanine p-nitroanilide	Nil	Nil	Nil
9.	Valine-Phenylalanine-Lysine p-nitroanilide	Nil	Nil	Nil

The purified ISP1 was also analyzed by native polyacrylamide gel electrophoresis run under non-reducing and non-denaturing conditions. Only one major band was observed (Figure 1F). As mentioned above, we were also able to purify rISP1 using a Hitrap Benzamidine affinity chromatography column, to yield a single coomassie-stained band upon electrophoresis that co-migrated with ISP1 immunoreactivity on a western blot (Fig. 1C, Lane 5 and Fig. 1E for the western blot detection). However the protein recovered from the benzamidine column was enzymatically inactive and we believe that the column elution conditions denatured the protein. Thus, the affinity-purified enzyme could not be used for functional studies. However, in accord with the gels and ABP western blot data shown in Figure 1, the combined ion-exchange/gel filtration purification procedure yielded a homogenously pure ISP1 preparation that generated only a single biotin-labelled band when reacted with the activity based serine proteinase probe, biotinyl-linker-Pro-Lys-diphenylphosphonate (Bio-PK-DPP 4) [23]–[25] (Figure 1G, Lane 2). Thus, the enzyme preparation obtained from the gel filtration column, containing a single enzymatically active proteinase, was of sufficient purity (180 U/mg) to be used for all further studies. The identity of the purified enzyme was also confirmed by Mass Spectrometry (LC-MS/MS) from Institute of Biodiagnostics (IBD), University of Alberta (Data S1).

### Enzyme activity measurements with different substrates and inhibitors

Various chromogenic peptide substrates conjugated with p-nitroanilide/4-methoxy naphthylamide were used to characterize the catalytic nature of rISP1 (Table 1). Amongst the different substrates tested, only the ones having an Arginine/Lysine at the P1 position were hydrolyzed, namely N-Benzoyl-L- arginine p-nitroanilide hydrochloride (BAPNA), N-CBZ-arginine-arginine-arginine-4-methoxy naphthylamide (RRRmN), N-Benzoyl-isoleucine-phenylalanine-lysine p-nitroanilide hydrochloride (IFK-pNA) and N-Benzoyl-phenylalanine-valine-arginine p-nitroanilide hydrochloride (FVR-pNA). Towards IFK-pNA, ISP1 showed highest Km (9.6 mM) and hence the lowest affinity followed by BAPNA (Km = 0.61 mM), FVR-pNA (Km = 0.36 mM) and RRRmN (Km = ∼0.1 mM). The kcat and kcat/Km values are also shown in Table 1. Interestingly no enzyme activity was observed with H-D-valine-leucine-lysine p-nitroanilide hydrochloride (VLK-pNA) N(p-tosyl)-glycine-proline-lysine p-nitroanilide acetate (GPK-pNA) and Valine-Phenylalanine-Lysine p-nitroanilide (VFK-pNA). Similarly no reaction was observed upon using Alanine p-nitroanilide (A-pNA) and N-Succinyl-alanine-alanine-alanine p-nitroanilide (AAA-pNA).

In order to validate the results obtained with small chromogenic substrates and to identify specific inhibitors, many general serine protease inhibitors were tested employing 2 mM BAPNA as well as 150 µM VPR-AMC as substrate (Table 2). As expected, PMSF, α-1-Antitrypsin, Chymostatin, Gabexate mesylate and Soybean Trypsin Inhibitor (STI) and TLCK inhibited the enzyme activity of rISP1. Benzamidine hydrochloride, Leupeptin, Aprotinin and recombinant human Secretory Leukocyte Protease Inhibitor (rhSLPI) showed no noticeable effect.

**Table 2:** Dissociation constants (Apparent Ki) of general serine proteinase inhibitors for a single substrate reaction of recombinant ISP1.

	Inhibitor	Substrate	Ki (µM)
1.	PMSF	VPR-AMC (0.15 mM)	340
2.	α-1-Antitrypsin	BAPNA (2 mM)	2.0
3.	Chymostatin	BAPNA (2 mM)	220
4.	Gabexate mesylate	BAPNA (2 mM)	1700
5.	Soybean Trypsin Inhibitor	BAPNA (2 mM)	2.4
6.	Benzamidine Hydrochloride	BAPNA (2 mM)	No inhibition
7.	Leupeptin	BAPNA (2 mM)	No inhibition
8.	TLCK	VPR-AMC (0.15 mM)	30
9.	Aprotinin	BAPNA (2 mM)	No inhibition
10.	Recombinant human SLPI	BAPNA (2 mM)	No inhibition

### Substrate specificity determination of ISP 1 with phage display

2Having observed trypsin-like substrate specificity with small chromogenic substrates, we next turned to the phage-display approach, as outlined in Experimental Procedures, to assess ISP1 substrate specificity. The non-aligned unbiased amino acid sequences deduced from the DNA sequences of the random hexameric insert region from 24 plaques obtained from the panning procedure are listed in Table 3. The data show that out of 24 plaque sequences, 20 (83%) had at least one arginine/lysine residue in their sequences (Table 3, bold italic letters).

**Table 3:** Plaque sequences of random hexamer region obtained after biopanning with recombinant ISP1.

Plaque No.	AA1	AA2	AA3	AA4	AA5	AA6
1.	P	S	T	**R**	**K**	**K**
2.	T	G	G	W	**R**	F
3.	Q	W	P	M	L	V
4.	W	S	G	Q	D	I
5.	Y	**R**	**R**	G	H	L
6.	**R**	Y	**R**	T	S	L
7.	**R**	Y	**R**	T	S	L
8.	S	W	G	**R**	**R**	G
9.	V	**R**	**K**	V	A	A
10.	V	**R**	**K**	V	A	A
11.	T	G	G	W	**R**	F
12.	S	**R**	S	C	T	A
13.	L	E	A	L	V	V
14.	S	A	C	L	T	G
15.	C	L	**K**	T	E	G
16.	F	**R**	**R**	P	G	G
17.	**R**	G	W	G	T	V
18.	L	**R**	Y	V	T	Y
19.	G	G	**R**	V	V	T
20.	**R**	V	Y	L	L	S
21.	**R**	A	Q	M	G	E
22.	P	V	Q	**K**	**K**	H
23.	V	G	**R**	H	L	A
24.	**K**	G	W	E	G	C

**Figure 2:**
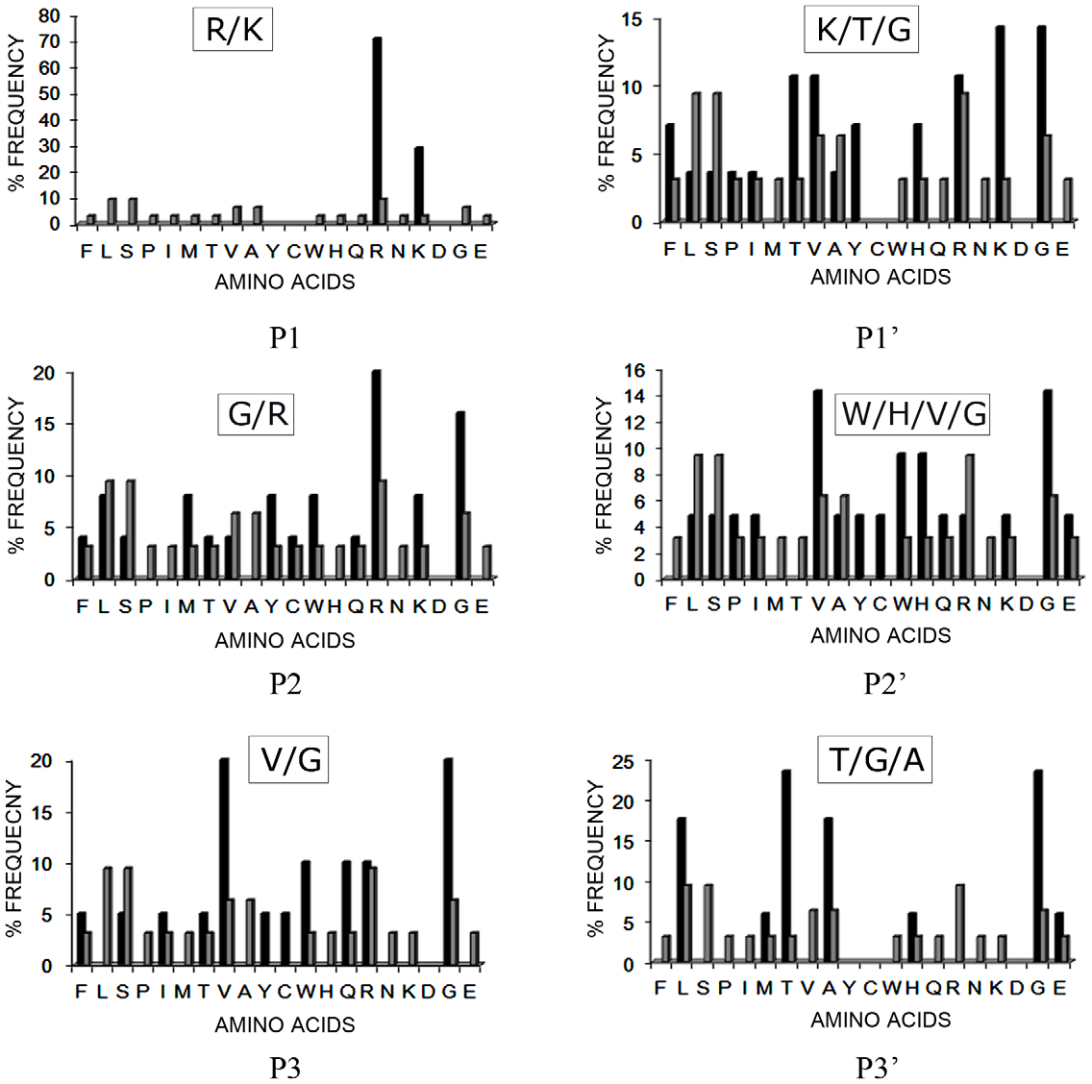
Biopanning results of T7 bacteriophage-displayed library of random peptides (6-mer) with purified ISP1. The frequency of occurrence of amino acid residues at a given position (P1, P2, P3, P1′, P2′ and P3′) after alignments assuming arginine/lysine at the P1 position (% frequency, y-axis) is shown. The observed frequencies (black bars) are shown in comparison to the frequencies expected in the absence of any bias (grey bars).

The biopanning data presented here validate in an unbiased way, our findings with the small chromogenic substrates indicating a trypsin-like activity. The biopanning data also demonstrated the presence of arginine/lysine residues in 71% of the phage plaques. Accordingly, resulting plaque sequences were aligned assuming an arginine/lysine residue at the P1 position. Upon analysis, lysine was found to be highly preferred over arginine residue at P1 position. Ratio of Arginine **_(Observed/Expected)_**/Lysine **_(Observed/Expected)_** is 1.28 in comparison to the same ratio of 0.38 in case of ISP1-ISP2 hetero-dimer reported earlier [9]. The frequency of occurrence of an amino acid at a given position (i.e. P1, P1′, P2…..) in comparison with its random probability at that position is shown in Figure 2. Based on the above analysis, the following preferred recognition/cleavage sequence motif was identified for the recombinant ISP1:

**P3  P2  P1  P1′   P2′     P3′**

**V/G G/R R/K K/T/G W/H/V/G  T/G/A**

**Figure 3:**
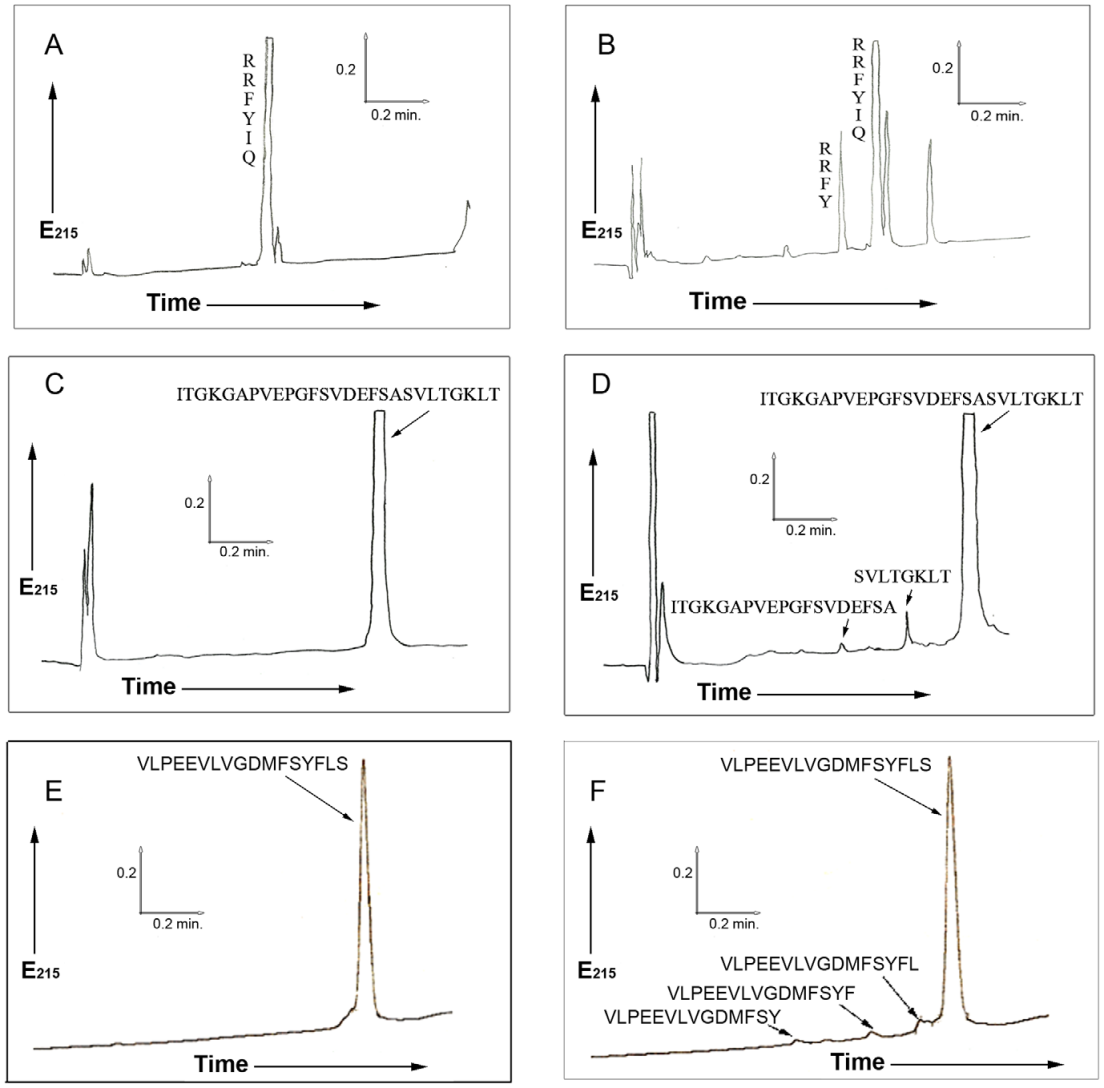
Cleavage of synthetic peptides in vitro by ISP1 and HPLC analysis of the peptide hydrolysis products followed by MALDI-TOF mass spectrometric analysis. The panels A–B represent the HPLC chromatograms of intact peptide –RRFYIQ-NH_2_ (A); and the peptide products after hydrolysis with ISP1 (B). The scales for time (minutes) and absorbance (E215: arbitrary absorbance units) are shown by the inserts (arrows) to the right of each chromatogram; (C) HPLC chromatogram of intact 27-mer peptide (ITGKGAPVEPGFSVDEFSASVLTGKLT-NH_2_) representing extracellular region of loop 1 in rat PAR_2_ downstream of the tethered ligand activating peptide (SLIGRL), (D) HPLC chromatogram showing cleavage products upon hydrolysis of the rat PAR_2_-derived 27-mer peptide sequence (above) after incubation with ISP1, as determined by MALDI-TOF spectrometry and amino acid analysis, (E) HPLC chromatogram of intact 17-mer peptide (VLPEEVLVGDMFSYFLS) representing extracellular region of loop 2 in rat PAR2, (F) HPLC chromatogram showing cleavage products upon hydrolysis of the rat PAR_2_-derived 17-mer peptide sequence (above) after incubation with ISP1, as determined by MALDI-TOF spectrometry and amino acid analysis.

As already mentioned these results validate the results presented above with synthetic chromogenic substrates and in addition demonstrate a preference for dibasic residues around the cleavage site at positions in the vicinity of cleavage site. To identify putative substrates of ISP1, the above generated consensus target amino acid sequence was compared with the NCBI non-redundant database in different permutations and combinations. A list of probable hits obtained with high degree of confidence is shown in Table 4, wherein PAR_2_ surfaced as a possible enzyme target. In order to validate our phage display results, we synthesized a number of peptides based on the above results. To facilitate the identification of the cleavage products by MALDI-TOF, the selected sequences were flanked by glycine residues: a) GGGGRGWGGG; b) GGGGKKGGGG; c) GGGGRKHGGG; d) GGVRKKVGGG. These peptides were subjected to cleavage *in vitro* with ISP1 as described in the methods section. To our surprise, although a number of these peptides showed some cleavage according to the HPLC analysis, we were unable to identify their sequences via MALDI-TOF analysis (data not shown). On the other hand, a target peptide (RRFYIQ) known to be cleaved by ISP2 (data not shown) was also cleaved by ISP1. The major ISP1 cleavage site of this peptide was found to be at a tyrosine (P1) residue (Figure 3A–B). As expected from our ISP2 cleavage data (data not shown), in the same experiment, ISP1 also yielded another product showing cleavage at an arginine (P1) as detected upon the direct electrospray (ES)-MS analysis of the reaction mixture (Figure S2). Thus ISP1 displayed both tryptic and chymotryptic activity in cleaving the peptide, RRFYIQ. We suspect that the multiple glycines flanking the other putative hydrolysis target sequence may have prevented cleavage.

**Table 4:** Potential putative substrates (mouse proteins) of ISP1.

	Putative Substrate	Accession no.	Cleavage motif
1.	Bone morphogenetic protein 1	NP_033885	195 GRRGGG 200
2.	Similar to aquaporin 5	XP_001476562	110 GRRGLG 115
3.	Procollagen, type VII, alpha 1	NP_031764	1526 GRRG 1529
4.	Procollagen, type XIX, alpha 1	NP_031759	406 GRRG 409
5.	Proteinase activated receptor 2	CAE11955	52 GKGV 55
6.	Prepro PC6	BAA02143	301 GRRGLG 306
7.	Voltage-gated potassium channel subunit Kv6.2 (Cardiac potassium channel subunit)	XP_140499	19 GRRGRG 24

### Cleavage of PAR-related sequences and silencing of proteinase activated receptors (PAR1, PAR2 and PAR4) with ISP1

#### Cleavage of synthetic peptides representing PAR_2_

Since PAR_2_ appeared in the list of putative substrates for ISP1 (Table 4), we synthesised the rat PAR_2_ N-terminal extracellular domain sequence (amino acid residues 48 to 71) that contains the GKG target sequence identified by our search to assess its hydrolysis by ISP1. We also synthesized the rat PAR_2_ extracellular loop 2 domain sequence (amino acid residues 229 to 245) in order to investigate its cleavage by ISP1. Cleavage of this domain would prevent receptor activation either by the activating peptide (SLIGRL-NH_2_) or trypsin. This rat receptor sequence is highly homologous with the human PAR_2_ sequence. Further, as in previous work [22], we evaluated the ability of ISP1 to cleave a sequence representing the cleavage/activation site of rat PAR_2_ (^30^ GPNSKGR/ SLIGRLDT ^45^P: tethered ligand sequence, underlined). Although the cleavage/activation sequence of PAR_2_ was not cleaved by ISP1 (data not shown), the 27 mer peptide (I^48^ TGKGAPVEPGFSVDEFSASVLTGKLT ^74^-NH_2_), representing the N-terminal extracellular domain of rat PAR_2_, downstream of the tethered ligand sequence, S^37^ LIGRLDTPPP ^47^, was successfully cleaved, as shown in Figure 3C–D. The cleavage products were identified by MALDI-TOF as well by amino acid analysis (SVLTGKLT-NH_2_ and ITGKGAPVEPGFSVDEFSA-NH_2_). Interestingly the cleavage takes place between an alanine (P1 sub site) residue and a serine (P1′ sub site) residue. These results suggest an elastase-like activity of ISP1 which has not been reported earlier. Similarly the 17-mer synthetic peptide derived from the extracellular loop 2 domain of rat PAR_2_ was successfully cleaved as shown in Figure 3E–F. The cleavage products identified by MALDI-TOF showed that the cleavage takes place at multiple sites namely between a tyrosine (P1 sub site) residue and a phenylalanine (P1′ sub site) residue; a phenylalanine (P1 sub site) and a leucine (P1′ sub site) residue; a leucine (P1 sub site) and a serine (P1′ sub site) residue. All these cleavage sites represent a chymotrypsin-like activity of ISP1.

#### ISP1-mediated release of N-terminal PAR sequences from cell-expressed receptors

**Figure 4:**
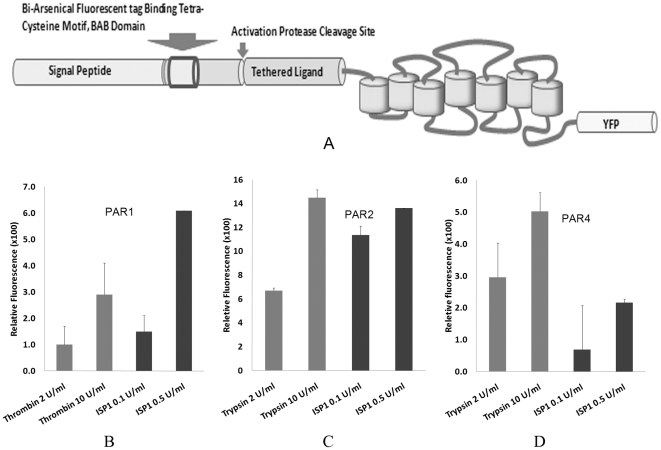
Measurement of the proteolytic ISP1-triggered release of the N-terminal biarsenical fluorochrome binding motif from PARs 1, 2 and 4 over-expressed on the cell surface of COS-1 cells. (A) a cartoon showing the details of biarsenical fluorochrome binding motif-tagged PARs; (B) Cleavage and release of the N-terminal PAR_1_-BAB motif; (C) Cleavage and release of the N-terminal PAR_2_-BAB motif; (D) Cleavage and release of the N-terminal PAR_4_-BAB motif; Trypsin and Thrombin are used as ‘positive control’ control proteinases in this experiment. The release of the N-terminal receptor BAB-containing domains was expressed in arbitrary fluorescence units, relative to the background fluorescence released by cells in the absence of proteinases [Relative Fluorescence = (signal with enzyme – signal in the absence of enzyme)/signal in the absence of enzyme).

In order to study the effects of ISP1 on intact cell-expressed PARs, we examined the ability of ISP1 to release the N-terminal domains of recombinant human PAR_1_, PAR_2_ and PAR_4_ expressed in a COS cell background. As outlined in Methods, the biarsenical binding, BAB site tag, was introduced at the N-terminus region of each PAR. The ability of ISP1 to release the PAR N-terminal BAB domains from the PARs, relative to trypsin (for PAR_2_) and thrombin (for PARs 1 and 4) as positive controls, is shown in Figure 4. The reaction was performed at 37°C for 30 minutes. Relatively low concentrations of ISP1 such as 0.1 and 0.5 units per ml showed significant concentration-dependent PAR cleavage activity for all 3 PARs. These results confirm that the N-terminal domains of PARs 1, 2 and 4 can be targets for ISP1 cleavage.

#### Disarming of PAR function by ISP1

**Figure 5:**
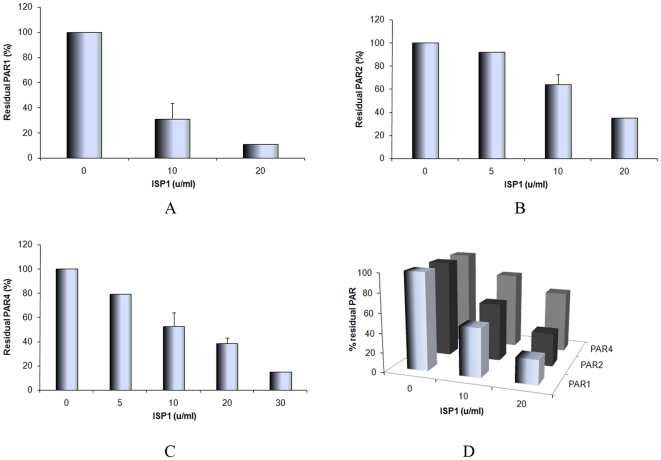
The disarming/inhibition of proteinase activated receptors (PARs) by ISP1 determined by a Calcium signaling assay. (A) Concentration-dependent disarming of human PAR1 is shown by measuring % residual PAR activation in response to the addition of Thrombin (0.5 units/ml) after pre-treating cells with increasing concentrations of ISP1, (B) Concentration-dependent disarming of rat PAR_2_ is shown by measuring the % residual PAR activation in response to the addition of Trypsin (1.0 units/ml) after pre-treating cells with increasing concentrations of ISP1, (C) Concentration-dependent disarming of rat PAR_4_ is shown by measuring the % residual PAR activation in response to the addition of Thrombin (0.5 units/ml) after pre-treating cells with increasing concentrations of ISP1 (D) Concentration-dependent inactivation of PAR_1_, PAR_2_ and PAR_4_ is shown by measuring the % residual PAR activation in response to the addition of the PAR-activating peptides, TFLLR-NH_2_ (PAR_1_AP, 5 µM), SLIGR-NH_2_ (PAR_2_AP, 10 µM) and AYPGKF-NH_2_ (PAR_4_AP, 100 µM) respectively after pre-treating cells with increasing concentrations of ISP1.

The ability of ISP1 to affect PAR function was evaluated with calcium signaling experiments using (a) cell lines over-expressing one or other of PARs 1 & 2 (as explained in Data S1) and (b) rat platelets that express only functional PAR_4_ but neither PAR_1_ nor PAR_2_. The results obtained are shown in Figure 5. While rISP1 did not activate a calcium signal via any of the PARs tested, the data (Figure 5A–C) clearly demonstrate the concentration–dependent disarming of PAR_1_, PAR_2_ and PAR_4_ by recombinant ISP1. Thus, pre-treatment of PAR-expressing cells with ISP1 markedly attenuated PAR signaling by the subsequent stimulation of the cells with an appropriate activating proteinase (thrombin for PARs 1 and 4; trypsin for PAR_2_). The results obtained in experiments wherein the cells were washed free of ISP1 prior to a challenge with a PAR-activating enzyme were equivalent to data from experiments wherein the cells were not washed and the PAR-activating enzyme was added directly to the ISP1-treated cells. Thus, an effect of ISP1 on the enzyme activity of either thrombin or trypsin was ruled out.

PAR_1_ signaling was attenuated to a larger extent by a low concentration (5 Units/ml) of ISP1 (20% residual PAR_1_ activity) in comparison with PAR_2_ (∼80% residual PAR_2_ activity triggered by trypsin after ISP1 pre-treatment) and PAR_4_ (∼75% residual PAR_4_ activity triggered by thrombin after ISP1 pre-treatment). Higher concentrations of rISP1 (20 units ISP1/ml) were able to eliminate PAR_1_ activity completely, while in comparison, PAR_2_ and PAR_4_ activities were reduced to ∼40% and ∼20% of control respectively.

In addition to diminishing proteinase-mediated activation of the PARs, pre-treatment of cells with ISP1 also diminished the cellular response to the PAR-activating peptides (TFLLR-NH_2_ for PAR_1_, SLIGRL-NH_2_ for PAR_2_, and AYPGKF-NH_2_ for PAR_4_). The ability of the PAR-activating peptides to activate PARs was diminished by ISP1 in a concentration-dependent manner as shown in Figure 5D. The effect on PAR_1_ was most highly pronounced in comparison with PAR_2_ and PAR_4_ after pre-treatment with the same concentration of ISP1 in the reaction milieu. These data reflected the results shown in Figure 5A–C. Thus, in addition to disarming the PARs to prevent enzyme activation, ISP1 was also able to inactivate receptor signaling via the PAR-activating peptides, presumably by cleaving one or more of the extracellular loops of the receptors, where the peptides dock to trigger a signal. This presumption is substantiated by our data demonstrating the ISP1-mediated cleavage of the 17-mer synthetic peptide representing extracellular loop 2 of PAR_2_ (Figure 3E–F).

### PAR-mediated ERK/MAP kinase activation

In order to explore the effect of ISP1 on the alternate routes of PAR signaling, we performed the experiments as per Ramachandran et al, 2009 [26] who showed a SLIGRL-NH_2_/PAR_2_-triggered activation of ERK/MAPkinase in PAR_2_-expressing KNRK cells. No change in phosphorylation status of ERK via MAP kinase activation was observed upon incubation of rat PAR_2_-expressing KNRK cells with ISP1 (Figure S3). Similar results were obtained with human PAR2 expressing cells.

## Discussion

### ISP1 is expressed as a monomer and doesn't form homo-dimers

Here we report the expression, purification, biochemical characterization and assessment of the impact on PAR signaling of recombinant implantation serine proteinase 1 (ISP1) produced by the methylotrophic yeast, *Pichia pastoris*. The main finding of our study was that the *Pichia* expression system yields a single enzymatically active monomeric form of ISP1 that can be covalently labelled with the biotinylated activity-based serine proteinase probe, BioPK-DPP4. Surprisingly, although our preliminary work with small chromogenic substrates predicted a trypsin-like substrate profile, the several approaches we used to assess substrate selectivity demonstrated that ISP1 displays a ‘promiscuous’ substrate specificity, targeting non-basic as well as basic amino acids in the P1 position. The expression profile for rISP1 demonstrated a typical expression pattern for a recombinant protein expressed in *Pichia*. rISP1 expression was observed within hours of starting the methanol feed and peaked after approximately 50 hours. While the *Pichia* expression system is notorious for expressing different protein isoforms with varying degrees of glycosylation, in our work, only a single enzymatically active species was expressed in a ∼34 kDa form, and not as a dimer. Since native ISP1 from uterine endometrium and embryos has been found to exist only in a hetero-dimeric form together with ISP2 [2], it is interesting that we did not detect recombinant ISP1 as a homo-dimeric species.

The affinity chromatography (benzamidine column) was not able to yield active enzyme, presumably because of the lability of the enzyme under conditions of column elution. Notwithstanding, the conventional purification approach involving ion exchange chromatography followed by gel filtration resulted in a substantial purification in terms of specific activity and the presence of only one coomassie blue stained as well as ABP-labelled product (Figure 1 and Data S1). The fact that we used a ‘proteinase free’ *Pichia pastoris* strain (SMD1168) to express a recombinant ISP1, provides more confidence in using the purified enzyme preparation for further studies such as substrate specificity determination and functional assays.

This purified preparation was used for characterizing the biochemical nature of ISP1 employing a limited number of synthetic low molecular weight chromogenic substrates and inhibitors. Based on its apparent K_m,_ kcat and kcat/Km ratio for substrates having an arginine/lysine at P1 position (Table 1), we confirmed that rISP1 possesses ‘trypsin like’ proteolytic activity, similar to its native counterpart i.e. the ISP1–ISP2 hetero-dimer [2]. Whereas the ISP1–ISP2 hetero-dimer showed a preference for arginine over lysine at the P1 position, recombinant ISP1 appeared to prefer lysine at the P1 position based upon results obtained with phage display peptide mimetics. The inhibition profile obtained with a number of serine proteinase inhibitors was in common with the profile obtained using the ISP1–ISP2 hetero-dimer isolated from natural sources [2]. PMSF, TLCK, α-1 antitrypsin, chymostatin, gabexate mesylate and trypsin soybean inhibitor (TSI) all showed a strong inhibitory activity against monomeric rISP1.

### ISP1 is a serine proteinase with mixed substrate specificity

Using phage display peptide mimetics, we observed that recombinant ISP1 doesn't observe strict substrate specificity norms as might have been expected from the preliminary results with the small substrates. Interestingly, we detected cleavage not only at P1 arginine residues as expected but also at P1 tyrosine and phenylalanine residues (Figure 3B and 3F), showing that ISP1 exhibits both chymotrypsin-like as well as trypsin-like specificity. Using the phage-displayed library, we did not detect any elastase-like activity (cleavage with glycine/alanine/valine at the P1 position) in these experiments. But the data obtained from cleavage of rat PAR_2_ peptide (27 mer) demonstrates the modest but unequivocal elastase-like cleavage specificity of ISP1 (Figure 3C–D). These results demonstrate that no single method is sufficient to obtain complete substrate specificity data of a proteolytic enzyme. However, upon putting together all the results obtained with chromogenic substrates, inhibitors, phage-display, and *in vitro* cleavage studies, it can be said that recombinant ISP1 is promiscuous and displays mixed substrate specificity *in vitro*. Although the importance of conserved S1 sub site (T_216_ and V_226_) has been emphasized in case human elastases [27], in mouse elastases no such conserved residues have been found. The ISP1 sequence also does not possess this conserved S1 sub site.

Of note, catalytic promiscuity is widespread amongst enzymes, especially amongst detoxifying enzymes like cytochrome P 450s, Glutathione S-tranferases and other species [28], [29]. In the case of proteinases, as well, target promiscuity has been well documented [30]. For instance, amongst the eight different proteases evaluated in an individual study, Cruzain and Papain were found to be very highly promiscuous, cleaving a variety of targets, in comparison with very restricted target specificity in the case of Granzyme B [30]. Amongst serine proteinases, human neutrophil elastase was found to be the most promiscuous, followed by chymotrypsin, plasmin, trypsin and thrombin in that order.

A distinct structure-activity relationship has previously been seen with human lung tryptase, which exists in both monomeric and tetrameric forms [31]. Similar studies done with human β-tryptase [32] and granzyme A [33] have demonstrated that the recombinant proteinases expressed in monomeric/multimeric forms show diverse and expanded substrate specificity in comparison to their native multimeric counterparts. Different reasons have been suggested for the increased substrate promiscuity exhibited by monomeric as well as dimeric proteases in comparison to tetrameric ones. However, ours is the first report describing a change in the specificity of a proteolytic enzyme by virtue of its existence in a monomeric versus dimeric form. Further work is being done to express and purify recombinant ISP2 produced by its heterologous expression in *Pichia pastoris* so as to characterize its substrate specificity and to attempt to generate active ISP1/ISP2 hetero-dimers. In addition, we will try to obtain ISP1/ISP2 knock-outs and then study the other enzyme. Hence, a more complete picture should emerge about this structure-activity relationship amongst the monomeric recombinant forms and the hetero-dimeric form of the ISPs.

### ISP1 can disarm and inactivate proteinase activated receptors in a concentration-dependent manner

According to our preliminary data establishing a trypsin-like activity of ISP1 (above), we anticipated that like trypsin, ISP1 would activate PAR_2_ by cleavage at its R^36^/S^37^ bond to reveal the receptor-activating tethered ligand. Utilising a novel PAR construct expressing an N-terminal BAB domain that can be removed by proteolytic cleavage we indeed found that rISP1 can release the N-terminal BAB-containing sequence of PAR_2_ from the C-terminal portion of its extracellular domain. Similarly, ISP1 was able to release N-terminal fragments from PARs 1 and 4 (Figure 4). Surprisingly, however, ISP1 did not cleave the synthetic peptide representing the cleavage/activation sequence of rat PAR_2_ ((^30^ GPNSKGR/ SLIGRLDT ^45^P) either at the expected ^36^R/^37^S bond or at the ^41^R/^42^L position, as does trypsin. Nonetheless, a 27 mer peptide (I^48^ TGKGAPVEPGFSVDEFSASVLTGKLT ^74^-NH_2_), representing the N-terminal extracellular region of PAR_2_, C-terminal to the cleavage-activation sequence shown above, was proteolytically cleaved by ISP1. Interestingly recombinant ISP1 cleaves this PAR_2_ peptide at an alanine (P1) residue, representing an elastase-like activity, unlike its trypsin-like activity observed with simple chromogenic substrates with arginine/lysine at the P1 position (as described above). Trypsin-like activity was also found for the native ISP1–ISP2 hetero-dimer as determined by the phage display approach [9].

For the monomeric form of ISP1, however, our phage display data combined with the results of the cleavage of putative substrate peptides *in vitro*, including the PAR_2_ –derived peptide sequences, indicate that the enzyme displays very mixed substrate specificity, having a capability of acting as a trypsin-like, chymotrypsin-like as well as an elastase-like serine proteinase. This kind of multiple substrate specificity has been found for many serine proteinases viz. human kallikreins KLK14 [34], KLK6 [9] and neutrophil elastase.

One of the most significant findings of our study was that the functional signaling through human, rat and murine PARs can be regulated by ISP1 in a negative way. The results obtained clearly demonstrate that recombinant ISP1 in a concentration-dependent manner can suppress the activation of PAR_1_, PAR_2_ and PAR_4_ by their activating proteinases (thrombin and trypsin). Since ISP1 treatment of PAR-expressing cells also attenuated subsequent PAR activation by the agonist peptides, it would appear that ISP1 targets not only the N-terminal extracellular domain (release of the N-terminal tetracystein BAB moiety) but also the extracellular receptor loops responsible for mediating PAR agonist peptide signalling. This hypothesis was confirmed by the successful cleavage of 17-mer synthetic peptide derived from the extracellular loop 2 region of PAR_2_, as shown in Figure 3E–F. In a separate experiment it was found that ISP1 doesn't have any inhibitory effect on the enzyme activity of trypsin and thrombin (data not shown), another possible explanation for this phenomenon.

Proteinase activated receptors are known to be expressed in the uterine endometrium [35], [36], myometrium [37], endometrial stromal cells [38], cervix [39] and placenta [40]. They have been implicated in several functions in uterus including thrombin/trypsin induced myometrial contraction [37], endometriosis [36], pregnancy and labour [41] and remodelling of the endometrium [42]. The functional studies have confirmed that ISPs are important for the successful hatching of the embryo and its implantation in the uterine wall [2], [7]. But, their molecular mechanism of action is not yet resolved. Also the knock out studies can be performed in the murine model to check the role of ISP1 alone in the absence of ISP2. Based on the results obtained, we suggest that ISP1 may act on PARs *in vivo* to inhibit signaling through these receptors. Since both PAR_2_ as well as ISP are known to be expressed in the endometrium, this phenomenon may be important in processes such as endometrial tissue remodelling [42] and maintenance of pregnancy [43] where the PARs are implicated.

## Materials and Methods

### Molecular Cloning

PCR fragments were generated using Platinum Pfx DNA polymerase (M/S Invitrogen) from the Strypt (ISP1) clone previously generated in this lab [3]. Primers used to generate PCR products for cloning into the *Pichia* expression vector pICZ alpha B (M/S Stratagene) were as given below:

ISP1 forward: 5′-CGC GAA TTC ATT GTG GGG GGT CAA CGT ACC-3′

ISP1 reverse: 5′-ACG ACG GCG GCC GCC TGG ATG TGT TGA TGG AT-3′

The cloned construct was designed to start at the N-terminus with the amino acid sequence, IVGG… that represents the N-terminus of active tryptases. Thus, it was expected that the expressed enzyme would be secreted in an enzymatically active form. The resulting (744 bp) PCR product was inserted into the *Xho* I and *Eco*RI sites of pICZ alpha B flanked by nucleotide sequence for Myc tag as well as a (His)_6_ tag at the C-terminus. This plasmid was transfected into the protease deficient strain of *P. pastoris* (SMD1168). Selection was performed in YPDS (1% yeast extract, 2% peptone, 2% dextrose, 1 M Sorbitol, ±2% agar and 100 ug/ml Zeocin) medium.

### Expression Studies

Expression studies were performed in general as per ‘manufacturers’ instruction and as described in Data S1.

### Protein purification

The fermentation broth was centrifuged at 5000 g employing Beckman centrifuge (rotor JA-10). The supernatant was subjected to column chromatography using the Duo Flow FPLC system (Bio-Rad) as per Sharma et al, 2006 [2]. Protein purification involved sequential ion exchange (DEAE Sepharose) chromatography (step-gradient) and two gel filtration steps a) Superdex-200 and b) Superdex-75. Affinity chromatography was also evaluated using a nickel-containing NiNTA column and a HiTrap™ Benzamidine column (GE Healthcare). Further details pertaining to the isolation of ISP1 are found in the figure legends (Figure 1).

The initial attempts to purify the expressed ISP1 were problematic, since (a) the cMyc tag did not visualize on western blots and (b) affinity purification with either the nickel or benzamidine columns did not yield active enzyme, because of the non-functional (His)_6_ tag and column elution conditions respectively. Notwithstanding, the benzamidine column did yield highly purified protein (see Figure 1C, lane 5), albeit enzymatically inactive. Thus, for purifying active recombinant ISP1 we turned to the use of conventional ion exchange chromatography followed by gel filtration as was previously done for the purification of the native ISP complex from biological fluids [2].

Protein concentrations were determined using the Bio-Rad DC protein assay (modified Lowry's assay) kit as per manufacturer's instructions. ISP1 was monitored by immunoblotting using 10% PAGE under denaturing or non-denaturing conditions. Proteins were transferred to nitrocellulose membrane and probed with custom made anti-ISP1 monoclonal antibodies prepared by Immuno-precise Antibodies Ltd., Victoria, Canada. A horseradish peroxidase (HRP)-labelled anti-mouse IgG antibody (GE Healthcare) was used at a 1∶10,000 dilution to generate the chemiluminescence signal for detection on BioMax film™ (Kodak).

### Activity-based serine proteinase probe labelling of ISP1

To label ISP1 with an activity-based probe (ABP), we used a strategy previously described for the identification of serine proteinases using biotinylated diphenyl phosphonate probes [23]. Based on the synthesis of probes with proline-lysine or asparagine-lysine sequences in the P2 and P1 enzyme target positions [24], we elected to use the biotinylated probe with the PK sequence as the tryptic target (so-called, compound 4, Bio-PK-DPP 4). The procedure followed in our experiments was essentially as per Oikonomopoulou et al, 2008 [25], who used this same ABP to label human tissue kallikreins. The reaction of the ABP was done with ISP1 as well as trypsin (control) using varying concentrations of ABP and enzyme. Enzyme was reacted at room temperature with the ABP (100 µM final concentration of ABP dissolved in DMSO) in 50 mM Tris.HCl (pH 7.4) in the presence of 1.5 mM Calcium chloride and 0.2%(v/v) NP40. The reactions were done at room temperature for 1 hour. The reaction (total volume 20 µl) was terminated upon the addition of 20 µl of 2× electrophoresis sample buffer containing β-mercaptoethanol and reaction products were heat-denatured for 3 minutes at 92°C. The samples were resolved by SDS/PAGE, and transferred to poly-vinylidene difluoride (Hybond-P, Amersham Pharmacia, Piscataway, NJ, USA) membranes. Membranes were blocked with 2% ECL Advance blocking solution (GE Healthcare, CPK1075) and 0.1% Tween-20 in isotonic phosphate-buffered saline, pH 7.4 (PBST), washed in PBST, treated with VectaStain (Vector Laboratories Burlington, ON, Canada) in PBST, to detect ABP-biotinylated enzyme. Membranes were washed, treated with ECL Plus reagents (Amersham Pharmacia, Piscataway, NJ, USA), and ABP-biotinylated enzyme was detected using the Storm 860 machine (Amersham Biosciences, blue fluorescence mode) and Image Quant software (Amersham Biosciences).

### Enzyme Kinetics

p-Nitroanilide substrates, Valine-Proline-Arginine-Amino methyl coumarin (VPR-AMC), Benzamidine Hydrochloride, TLCK (N -p-tosyl-L-lysine chloromethyl ketone), APMSF (4-Amidino-Phenyl)-Methane-Sulfonyl Fluoride), Trypsin Soybean Inhibitor (TSI) and Gabexate mesylate were obtained from Sigma Aldrich and Co. Oakville, ON Canada. Chromogenic assays were performed using the p-Nitroanilide/methyl-naphthylamide conjugated peptidic substrates. The reaction conditions used were: 25 mM Tris.Cl (pH 8.0) buffer with 10 mM EDTA reaction buffer, in a total reaction volume of 0.5 mL including varying concentrations of the substrates and enzyme sample. The reactions were done at room temperature in a glass cuvette (1 cm path length), and scanned at wavelength of 405 nm (in case of p-nitroanilide conjugated substrates) and 540 nm (in case of methyl-naphthylamide substrates) using a spectrophotometer. 1 Unit of enzyme is defined as the amount of enzyme capable of the breakdown of 1 micromole of substrate/min.

The catalytic activity of recombinant ISP1 with various small chromogenic peptide substrates was characterized by measuring enzyme cleavage at differing substrate concentrations and plotting enzyme activity [V] vs. substrate concentration [S] and calculating Michaelis Menten constant (Km). The Km was calculated using the Michaelis Menten equation (Km = [S] at ½ Vmax). Sigma plot was used for plotting the values obtained and determining Michaelis Menten Constant (Km). Inhibition of proteolytic activity of recombinant ISP1 was determined by measuring enzyme activity in the presence of varying concentrations of serine proteinase inhibitors. 2 mM BAPNA and 150 uM VPR-AMC were employed for enzyme assays in these inhibition studies. Sigma plot was used for plotting the values obtained in order to determine dissociation constant (Ki). Enzyme concentration [E] used for these experiments was 6.5 uM. This value was employed for calculating turnover number (kcat) and enzyme efficiency represented by kcat/Km ratio.

### Substrate specificity determination by T7 phage display

These studies were done essentially as described by Sharma et al, 2008 [9]. The procedure is described in Data S1.

### *In vitro* cleavage of synthetic peptides and identification by HPLC-MS

Synthetic peptides (1 mg/ml) diluted in 25 mM Tris.Cl (pH 7.8) were incubated with 2.0 units of purified recombinant ISP1 (total volume of reaction mixture: 0.5 ml) at 37°C overnight. The reaction was terminated by the addition of 0.1% trifluoroacetic acid and the mixture was separated using HPLC with an acetonitrile gradient in 0.1% v/v trifluoroacetic acid. The positions of the hydrolysis products monitored by ultraviolet absorption (E_215_) were compared to the elution position of the intact peptide. The masses of the individual peaks (E_215_) were determined by MALDI-TOF mass spectroscopy and were used to deduce the predicted amino acid sequences, based on the parent peptide sequence (amino acids are abbreviated by their one-letter codes, e.g., A = alanine, R = arginine). Except where indicated, all chemical reagents were from VWR International (Mississauga, ON, Canada).

### Studies with Proteinase activated receptor 1, 2 and 4 (PAR_1_, PAR_2_ and PAR_4_)

The procedures followed were essentially those used by Oikonomopolou et al, 2006 [25] and as described in brief in Data S1.

### Mass Spectral Analysis of Peptide proteolysis products

The rat PAR_2_ sequence representing the cleavage/activation domain (^30^ GPNSKGR/ SLIGRLDT ^45^P: tethered ligand sequence, underlined), the 27-mer synthetic sequence derived from the N-terminal residues 43 to 74 of rat PAR_2_ (I^48^ TGKGAPVEPGFSVDEFSASVLTGKLT ^74^) and a 17-mer synthetic peptide sequence derived from extracellular loop 2 of rat PAR_2_ (V^229^ LPEEVLVGDMFSYFLS ^245^) were selected for the proteolysis studies. In the rat receptor, the ^48–74^PAR_2_ sequence occurs downstream from the revealed tethered ligand sequence (S^37^ LIGRL ^42^ —) and C-terminal to a series of three prolines that would render the domain exposed to proteinases. The 17-mer ^229–245^PAR_2_ sequence is the potential binding site of the activating peptide. Comparable sequences are present in human PAR_2_. These peptides at a concentration of 100 µM were incubated with implantation serine proteinase 1 for varying times up to overnight at 37°C. The incubation buffers were identical to the buffers used for the measurements of enzymatic activities using AMC-substituted peptide substrates. Reactions were terminated either by rapid freezing in liquid nitrogen or by the addition to the proteolysis sample of 2 volumes of a “stop solution” comprising 50% acetonitrile and 0.1% trifluoroacetic acid in water. Samples were either subjected immediately to HPLC analysis, with collection of the E_215_ peak fractions, or were stored at −80°C for further processing. The cleavage products in peaks of absorption resolved by HPLC were quantified (E_215_), collected and their identity was established by mass spectral MALDI analysis. Some of the peaks recovered from the HPLC columns, but not ascertained by mass spectral analysis (MALDI-TOF), were identified by total amino acid analysis. Analyses were done at the Southern Alberta Mass Spectrometry Facility at the University of Calgary, Faculty of Medicine (Calgary, Alberta, Canada).

### Evaluation of activation of P44/42 MAP Kinase Signaling by ISP1

Western blot detection of P44/42 ERK was performed essentially as described [26]. In brief, KNRK cells [25] that had been transfected with the PAR_2_ expressing construct (PAR_2_ -KNRK) or empty vector (pcDNA3-KNRK) were serum starved and stimulated with ISP1 for 10 min. Cells were placed on ice and agonists were removed prior to lysis with cold lysis buffer (Roche). Protein samples were boiled for 10 min in Laemelis buffer and resolved on SDS-PAGE gels. Activation of MAP Kinase was monitored by immunoblotting with phospho-specific ERK antibodies (Cell signaling) and relative increases in ERK phosphorylation was quantified relative to total ERK signal detected in the same samples. As a positive control cells were activated with SLIGRL-NH_2_ that causes a PAR_2_-mediated increase in MAPkinase phosphorylation.

## Supporting Information

Data S1 **Supplementary data.** a) Expression Studies b) Substrate specificity determination by T7 phage display c) Studies with Proteinase Activated Receptors (PAR_1_, PAR_2_ and PAR_4_) d) Supplementary Results e) Supplementary Table 1 f) Mass Spectrometry Results for rISP1 g) Supplementary References.
(DOC)

Figure S1 **Expression of ISP1.** (A) Fermentation parameters including pH, temperature, % dissolved oxygen (DO) and packed cell volume (PCV) recorded during a typical fermentation run (10 litre working volume), (B) Feeding rates of glycerol, methanol and oxygen shown in % figures during a typical fermentation run (10 litre working volume), (C) Expression of recombinant ISP1 as determined by Western blot analysis using monoclonal anti-mouse ISP1 antibodies (samples were withdrawn at different time intervals), lane 1–30 hrs., lane 2–40 hrs., lane 3–50 hrs., lane 4–60 hrs., lane 5–70 hrs., lane 6–80 hrs., lane 7–90 hrs., lane 8–100 hrs.
(DOC)

Figure S2 **ES-MS analysis of reaction mixture showing the detection of FYIQ as a cleavage product of RRFYIQ when incubated with ISP1.**
(DOC)

Figure S3 **ERK activation assay with rat PAR_2_ and control (pcDNA) transfected KNRK cells.** No activation of ERK is observed upon incubation of cells with ISP1for 10 min.
(DOC)
