# Supplementary material for: Implantation Serine Proteinase 1 Exhibits Mixed Substrate Specificity that Silences Signaling via Proteinase-Activated Receptors
Source: PLoS One. 2011 Nov 23;6(11):e27888. doi: 10.1371/journal.pone.0027888 (PMC3223204; doi:10.1371/journal.pone.0027888)
Supplement: Data S1 — Supplementary data. a) Expression Studies b) Substrate specificity determination by T7 phage display c) Studies with Proteinase Activated Receptors (PAR1, PAR2 and PAR4) d) Supplementary Results e) Supplementary Table 1 f) Mass Spectrometry Results for rISP1 g) Supplementary References. (DOC) [file pone.0027888.s001.doc]

**EXPRESSION STUDIES**

**Shake flask fermentation**

Buffered Minimal Glycerol Yeast (BMGY) medium was inoculated from a single colony in a baffled flask and grown at 30^o^C in an incubating shaker (250-300 rpm) for 16-18 hours. Cells were centrifuged at 3000 x g for 15 min. The cell pellet was resuspended in BMGY medium (100mM potassium phosphate, pH 6.0, 1.34% YNB (Yeast Nitrogen base), 0.00004% Biotin and 1% glycerol) for inoculation of the main fermentation batch in baffled flasks. The main fermentation was performed in Buffered Minimal Methanol Yeast (BMMY) media (100mM potassium phosphate, pH 6.0, 1.34% YNB, 0.00004% Biotin and 0.5% Methanol). Cells were grown at 28^o^C at 250-300 rpm. 0.5 % methanol (100%) was fed every 24 hours. The batch was harvested by centrifugation (3000 x g for 15 min) after 72 hours of growth in the induction (Methanol-fed) phase.

**Scale-up Fermentation**

Scale-up studies were performed in a BioFlow 110 (New Brunswick Scientific) autoclavable glass vessel fermentor (1.3L and 14L total volume) as per *Pichia* process fermentation guidelines developed by Invitrogen. The seed was grown in the shake flask using BMGY medium (as described above). 10% inoculums were used for seeding the main fermentation. The fermentation was carried out in Fermentation Basal Salts medium (26.7 ml of Phosphoric acid (85%), 0.93 g Calcium sulphate, 18.2 g Potassium sulphate, 14.9 g Magnesium sulfate.7H_2_O, 4.13 g Potassium hydroxide and 40 g Glycerol, 1.0 litre volume with water) with 4.35 ml PTM Trace salts /L medium (6 g Cupric sulfate.5H_2_O, 0.08 g Sodium iodide, 3 g Manganese sulfate.H_2_O, 0.2 g Sodium molybedate.2H_2_O, 0.02 g Boric acid, 0.5 g Cobalt chloride, 20 g Zinc chloride, 65 g Ferrous sulfate.7H_2_O, 0.2 g Biotin, and 5 ml Sulphuric acid per litre water) in three distinct phases namely; glycerol batch (18-24 hours), glycerol fed-batch (4 – 5 hours) and methanol fed-batch (~70 hours). Each fermentation-run lasted from 100 – 110 hours. Optimal conditions for growth and expression were maintained throughout the fermentation (30^o^C, pH 5.0, >20% dissolved oxygen). The pH was maintained by feeding of 28% ammonium sulphate solution.

In order to optimize the expression of recombinant ISP1 protein, expression studies were done at 1L and 10L working volume. The fermentation parameters viz. temperature, pH, and dissolved oxygen (Supplementary Figure 1a), glycerol/ethanol and ammonia feeding rate (Supplementary Figure 1b) were maintained as shown in supplementary figure 1. Although most of these parameters did not change significantly with the scale of operation, there was a higher demand for oxygen at the 10L scale (Supplementary Figure 1b) in order to maintain dissolved oxygen levels above 20%. In contrast, when fermentation was done at the 1.0L scale, the O_2_ feed rate did not go beyond 5% (data not shown).

**SUBSTRATE SPECIFICITY DETERMINATION BY T7 PHAGE DISPLAY**

**Construction of library, biopanning, selection and titration**

A library of random hexapeptides (6-mers) was generated as described by Karlson et al., 2002 [1] with minor modifications. The sequence of synthetic degenerate oligonucleotides inserted in the coding region of T7 phage capsid protein (employing T7 Select 1-1 vector arms, T7 Select system, Novagen Canada), encoding a random hexamer followed by (His)_6_ tag is shown here: 59-AAT TCT CTC ACT CCA GGC GGC-(NNK)_6_-GGT GGT CAT CAC CAT CAC CAT CAC TAA-39 (N represents any nucleotide and K represents T or G). The total library size was estimated to be 1.5x10^8^ pfu/ml based on number of transformants. Subsequently, the library was amplified to a titre of 1.0x10^10^ pfu/ml. In addition to confirming the randomness of the library, we also validated our biopanning approach using thrombin as a test enzyme with previously known substrate specificity. Biopanning was performed using either 2.0 units/ml of ISP1 enzyme complex [2] per cycle in 25 mM Tris.Cl, pH 8.0 (total volume of reaction mix: 0.5 ml) at 37^0^C overnight, subsequent to the binding of the amplified phage preparation (1x10^10^ pfu/ml) with 100 ul Ni-NTA agarose beads as described in Karlson et al., 2002 [3]. A control elution was performed using 500 mM Imidazole solution.

The process was repeated five times and the phage plaques obtained were amplified by lysing cultures of *E. coli* (BLT 5403). Amplified phage plaques from round five of biopanning were plated out on a top agarose plate and were picked up for subsequent amplification of DNA by PCR (using T7 primers). The PCR product was purified using QIAquick PCR purification kit (Qiagen, Mississauga, ON, Canada) and sequenced. The amino acid sequence of the random peptides displayed is deduced from the DNA sequence thus obtained. The samples of phage plaques obtained after every round of biopanning were serially diluted and plated onto LB (Amp+) Agar medium after mixing with suspension of *E. coli* (BLT5403) culture in 0.1 M MgSO4 and top agarose. The phage plaques growing on the plates were manually counted after incubating the plates for 3–4 h at 37^0^C.

**STUDIES WITH PROTEINASE ACTIVATED RECEPTORS 1, 2 AND 4 (PAR_1_, PAR_2_ & PAR_4_)**

**Peptides and other Reagents**

All peptides were synthesized by standard solid phase methods by the Peptide Synthesis Facility at the Faculty of Medicine, University of Calgary (peplab@ucalgary.ca). Peptide composition and purity (>95%) were ascertained by HPLC analysis, amino acid analysis, and mass spectrometry. Stock solutions (about 1 mM) were prepared in 25 mM HEPES buffer, pH 7.4, and peptide concentrations were verified by quantitative amino acid analysis. Porcine trypsin (cat. number T-3030; 14,500 units/mg) and the calcium ionophore A23187 were from Sigma-Aldrich. High activity human thrombin (cat. number 605195, lot B37722; 3,186 NIH units/mg) was from Calbiochem. The calcium indicator Fluo-3 acetoxymethyl ester was from Molecular Probes (Eugene OR).

**Cell culture and PAR-expressing cell lines**

The Kirsten virus-transformed normal rat kidney (KNRK) epithelial cell line expressing wild-type rat PAR_2_ (KNRKrPAR_2_), validated previously for studies of receptor activation [4] was used to evaluate the PAR_2_-regulating properties of the ISP1 by the methods described previously [5, 6]. Human embryonic kidney cells (HEK293) that express the SV40 T-antigen were kindly provided by Dr. Jonathan Lytton, University of Calgary, Faculty of Medicine, Calgary (Alberta) Canada. The human HEK293 cells, that constitutively express both PAR_1_ and PAR_2_, were grown under similar conditions as described for the KNRK cells, but in the absence of geneticin, as outlined for the PAR-mediated calcium signaling procedure described previously [6]. All cells were sub-cultured by dissociation in an isotonic EDTA/saline solution, pH 7.4, without the use of trypsin, to avoid cleavage of PARs.

**Measurement of the release of the N-terminal domains of PARs 1, 2 and 4 by ISP1 using biarsenical fluorochrome binding motif-tagged receptors**

The biarsenical fluorochrome binding domain (BAB), containing a tetra-cysteine motif [7], FLNCCPGCCMEPA for PAR_1_, AFLNCCPGCCMEP for PAR_2_ and FLNCCPGCCMEP for PAR_4_ was inserted into the receptor N-terminal domains by introducing corresponding oligonucleotides just downstream of the signal peptide of PARs, after A_26_ for human PAR_1_, Q_27_ for human PAR_2_ and V_25_ for human PAR_4_. The PAR DNA constructs containing the N-terminal BAB domain and a YFP C-terminal fluorochrome tag were cloned in pCDNA3.1+ at the restriction enzyme sites between Eco RI and Xba I. COS-1 cells obtained from ATCC (Manassas, VA USA) were transected with the PAR constructs with the FuGENE6 transfection reagent (Roche Applied Bioscience). The expression level of the expressed receptor was quantified using a fluorescence plate reader, (Victor 4X, Perkin-Elmer), and confocal microscopy was used to monitor the receptor location by visualizing the receptor YFP tag. To detect ISP1, trypsin and thrombin-mediated proteolytic cleavage of the PARs and release of their N-terminal BAB-binding motifs, receptor-expressing cells were labeled with 0.2 μM of the bi-arsenical fluorochrome ReAsH (Invitrogen, Carslsbad CA, USA) together with proteinases for 30 min in HEPES-buffered saline pH 7.4, containing 1.5 mM CaCl_2_. Then, the peptide(s) containing the receptor BAB tetracysteine motif released by proteinases from the receptor N-terminus into the cell culture supernatant was quantified using a Victor X4 fluorescent plate reader with an excitation wavelength of 540 nm and an emission wavelength monitored at 615 nm. The release of the N-terminal receptor BAB-containing domains was expressed in arbitrary fluorescence units, relative to the background fluorescence released by cells in the absence of proteinases [Relative Fluorescence = (signal with enzyme – background signal without enzyme)/ signal without enzyme].

One unit of trypsin activity was defined as 1 BAEE unit using trypsin from porcine pancreas as a standard purchased from Sigma Aldrich (T0303; approx 14,500 U/mg) and one unit of ISP1 activity was defined according to the enzyme’s ability to release one micromole of free p-nitroanilide from n-benzoyl arginine p-nitroanilide (BAPNA) per minute.

**Measurements of PAR-stimulated Calcium Signaling**

Cell lines (grown to about 85% confluence and disaggregated with calcium-free isotonic phosphate-buffered saline containing 0.2 mM EDTA) and rat platelets (harvested from platelet-rich anti-coagulated plasma) were prepared and used as previously described [4]. Cells were incubated for 25 min at room temperature with Fluo-3 acetoxymethyl ester (final concentration, 22 µM) along with 0.4 mM sulfinpyrazone. Cells were washed twice by centrifugation (using the following buffer without calcium) and resuspended in the calcium signaling buffer: 150 mM NaCl, 3 mM KCl, 1.5 mM CaCl_2_, 20 mM HEPES, 10 mM dextrose, and 0.25 mM sulfinpyrazone, pH 7.4.

Fluorescence was measured at 24 °C with an excitation wavelength of 480 nm and an emission recorded at 530 nm using an Aminco Bowman Series 2 Luminescence spectrometer (ThermoSpectronic Model FA354, Spectronic Unicam, Rochester, NY). The fluorescence signals caused by the addition of test agonists (trypsin, PAR-activating peptide or rISP1, added to 2 ml of a cell suspension of ~3 x 10^5^ cells/ml) were compared with the fluorescence peak height yielded by replicate cell suspensions treated with 2 µM ionophore A23187. This concentration of A23187 was at the plateau of its concentration-response curve for a fluorescence response in Fluo-3-loaded cultured cells. Under these conditions, the calculated values for intracellular calcium in the HEK and KNRK cells were
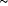
30 nM under basal conditions and about 340 nM upon exposure to A23187 [8, 9].

Previous work [6, 10] has shown that the fluorescence response of a cell preparation, as a percentage relative to the signal generated by 2 µM A23187, is a valid reference standard for the comparative determination of calcium signals for all PAR agonists. The cellular calcium response was therefore recorded as a percentage of the response generated by 2 µM ionophore A23187 [6]. Concentration-effect curves for recombinant enzyme (rISP1) were obtained by measuring the calcium signals generated by measuring the effects (receptor activation or disarming) of increasing enzyme concentrations in replicate cell suspensions (2 ml).

Receptor activation was monitored as an enzyme-stimulated increase in fluorescence emission (E530). As outlined in the following paragraph, receptor disarming or disabling was ascertained by the ability of ISP1 pre-treatment of the cells to abrogate the calcium signal caused by the subsequent addition of either a PAR-activating enzyme (thrombin for PARs 1 & 4; trypsin for PAR_2_) or a PAR-activating peptide (a reduced response to the activating peptide reflects receptor disabling). Values at each enzyme concentration represent the averages (±S.E: error bars above average values) of measurements done with at least three cell replicates derived from three or more independently grown cultures of PAR-expressing cells.

**Evaluation of Receptor Disarming/Inhibition Using the Calcium Signaling Assay**

Proteolytic removal of the tethered ligand by cleavage downstream from the activation domain can disarm a PAR, so as to inhibit its proteolytic activation but still leave the cell sensitive to activation by a PAR-activating peptide [6, 11, 12]. In this process, the proteinase amputates the receptor-activating sequence but may possibly also bind tightly in a non-catalytic mode to the receptor cleavage site, thereby inhibiting activation by other proteinases. To determine whether ISP1 can disarm/inhibit PAR_1_ for activation by thrombin, we exposed wild-type HEK cells for 10 min to increasing concentrations of ISPs, starting with ISP concentrations expected to result in a minimal activation of PAR_1_. In the continued presence of ISPs, the cells were then challenged either with a PAR_1_-activating concentration of thrombin (0.5 units/ml) or with a PAR_1_-activating concentration of the receptor-selective activating peptide, TFLLR-NH_2_ (5 µM). The thrombin-induced and peptide-induced calcium signals measured after ISP1 exposure were compared (% control) with the "control" calcium signal caused by these agonists in the absence of ISP1 exposure in the same cell preparation. The diminished "residual" PAR_1_ response to thrombin represents both disarming and desensitization.

As a variation on this protocol, cells first pre-treated or not with ISP1 for 10 min were washed free of enzyme and resuspended in calcium assay buffer for the measurement of enzyme (thrombin or trypsin)-activated and peptide-activated calcium signaling. This protocol ensured that the residual ISP1 in the incubation medium did not block or otherwise inhibit enzyme action. Thus, a reduced thrombin response in the washed, ISP1-treated cells, with retention of the signal caused by TFLLR-NH_2_, reflected a disarming of PAR_1_. Alternatively, a diminished response to the receptor-activating peptide after ISP1 treatment represents receptor desensitization/disabling and not necessarily disarming [6]. The same procedure was repeated to assess the disarming of rat PAR_2_ and rat PAR_4_ expressed in either KNRK cells (PAR_2_) or rat platelets (PAR_4_) respectively. Subsequent to incubation with increasing concentrations of ISP1, the cells were then challenged either (a) with a PAR_2_-activating concentration of trypsin (1.0 U/ml) / SLIGRL-NH_2_ (10 µM) or (b) with a PAR_4_-activating concentration of thrombin (0.25 U/ml) / AYPGKF (100 μM).

**SUPPLEMENTARY RESULTS**

Supplementary Table 1: Purification of recombinant Implantation serine proteinase 1

| **Sample** | **Volume (ml)** | **Protein Conc. (mg/ml)** | **Enzyme activity (U/ml)** | **Total Enzyme Activity Units** | **Specific activity (U/mg)** | **Fold Purification / step** |
| --- | --- | --- | --- | --- | --- | --- |
| Fermentation broth supernatant | *1000 | 3.5 | 1.9 | 1900 | 0.54 |  |
| DEAE Sepharose purified | 1000 | 4.5 | 9.4 | 940 | 2.1 | 3.9 |
| Superdex-75 purified | 500 | 0.55 | 100 | 500 | 180 | 87 |
|  |  |  | **Overall Yield (%)** | **26.3** | **Overall Folds of purification** | **340** |

**Mass Spectrometry Results for rISP1**

**
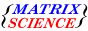
Mascot Search Results**

**Protein View**

1. Top of Form

Match to: **PRS28_MOUSE** Score: **119**

**Serine protease 28 OS=Mus musculus GN=Prss28 PE=1 SV=1**

Found in search of 5p065-uofc-1-30.mgf

Nominal mass (M_r_): **30609**; Calculated pI value: **8.70**

NCBI BLAST search of [PRS28_MOUSE](http://www.ncbi.nlm.nih.gov/blast/Blast.cgi?ALIGNMENTS=50&ALIGNMENT_VIEW=Pairwise&AUTO_FORMAT=Semiauto&CDD_SEARCH=on&CLIENT=web&COMPOSITION_BASED_STATISTICS=on&DATABASE=nr&DESCRIPTIONS=100&ENTREZ_QUERY=(none)&EXPECT=10&FILTER=L&FORMAT_BLOCK_ON_RESPAGE=None&FORMAT_OBJECT=Alignment&FORMAT_TYPE=HTML&GAPCOSTS=11+1&I_THRESH=0.001&LAYOUT=TwoWindows&MATRIX_NAME=BLOSUM62&NCBI_GI=on&PAGE=Proteins&PROGRAM=blastp&QUERY=MFRLLLLALSCLESTVFMASVSISRSKPVGIVGGQRTPPGKWPWQVSLRMYSYEVNSWVHICGGSIIHPQWILTAAHCIQSQDADPAVYRVQVGEVYLYKEQELLNISRIIIHPDYNDVSKRFDLALMQLTALLVTSTNVSPVSLPKDSSTFDSTDQCWLVGWGNLLQRVPLQPPYQLHEVKIPIQDNKSCKRAYRKKSSDEHKAVAIFDDMLCAGTSGRGPCFGDSGGPLVCWKSNKWIQVGVVSKGIDCSNNLPSIFSRVQSSLAWIHQHIQ&SERVICE=plain&SET_DEFAULTS.x=9&SET_DEFAULTS.y=5&SHOW_OVERVIEW=on&WORD_SIZE=3&END_OF_HTTPGET=Yes) against nr

Unformatted [sequence string](http://www.matrixscience.com/cgi/getseq.pl?SwissProt+PRS28_MOUSE+seq) for pasting into other applications

Taxonomy: [Mus musculus](http://www.ncbi.nlm.nih.gov/Taxonomy/Browser/wwwtax.cgi?lvl=0&id=10090)

Cleavage by Trypsin: cuts C-term side of KR unless next residue is P

Sequence Coverage: **13%**

Matched peptides shown in **Bold Red**

**1** MFRLLLLALS CLESTVFMAS VSISRSKPVG IVGGQRTPPG K**WPWQVSLR**M

**51** YSYEVNSWVH ICGGSIIHPQ WILTAAHCIQ SQDADPAVYR **VQVGEVYLYK**

**101** EQELLNISRI IIHPDYNDVS KRFDLALMQL TALLVTSTNV SPVSLPKDSS

**151** TFDSTDQCWL VGWGNLLQR**V PLQPPYQLHE VKIPIQDNK**S CKRAYRKKSS

**201** DEHKAVAIFD DMLCAGTSGR GPCFGDSGGP LVCWKSNKWI QVGVVSKGID

**251** CSNNLPSIFS RVQSSLAWIH QHIQ

  Residue Number  Increasing Mass  Decreasing Mass

**Start - End Observed Mr(expt) Mr(calc) Delta Miss Sequence**

**42 - 49 536.2273 1070.4399 1070.5661 -0.1262 0 K.WPWQVSLR.M**  ([Ions score 32](http://www.matrixscience.com/cgi/peptide_view.pl?file=../data/20100928/FteTlzESe.dat&query=10&hit=1&index=PRS28_MOUSE&px=1&section=5&ave_thresh=31&_ignoreionsscorebelow=0&report=0&_sigthreshold=0.05&_msresflags=1089&_msresflags2=2&percolate=-1&percolate_rt=0))

**42 - 49 536.2475 1070.4805 1070.5661 -0.0856 0 K.WPWQVSLR.M**  ([Ions score 20](http://www.matrixscience.com/cgi/peptide_view.pl?file=../data/20100928/FteTlzESe.dat&query=11&hit=1&index=PRS28_MOUSE&px=1&section=5&ave_thresh=31&_ignoreionsscorebelow=0&report=0&_sigthreshold=0.05&_msresflags=1089&_msresflags2=2&percolate=-1&percolate_rt=0))

**91 - 100 599.3064 1196.5983 1196.6441 -0.0458 0 R.VQVGEVYLYK.E**  ([Ions score 26](http://www.matrixscience.com/cgi/peptide_view.pl?file=../data/20100928/FteTlzESe.dat&query=16&hit=1&index=PRS28_MOUSE&px=1&section=5&ave_thresh=31&_ignoreionsscorebelow=0&report=0&_sigthreshold=0.05&_msresflags=1089&_msresflags2=2&percolate=-1&percolate_rt=0))

**91 - 100 599.3076 1196.6007 1196.6441 -0.0434 0 R.VQVGEVYLYK.E**  ([Ions score 39](http://www.matrixscience.com/cgi/peptide_view.pl?file=../data/20100928/FteTlzESe.dat&query=17&hit=1&index=PRS28_MOUSE&px=1&section=5&ave_thresh=31&_ignoreionsscorebelow=0&report=0&_sigthreshold=0.05&_msresflags=1089&_msresflags2=2&percolate=-1&percolate_rt=0))

**91 - 100 599.3194 1196.6243 1196.6441 -0.0199 0 R.VQVGEVYLYK.E**  ([Ions score 30](http://www.matrixscience.com/cgi/peptide_view.pl?file=../data/20100928/FteTlzESe.dat&query=18&hit=1&index=PRS28_MOUSE&px=1&section=5&ave_thresh=31&_ignoreionsscorebelow=0&report=0&_sigthreshold=0.05&_msresflags=1089&_msresflags2=2&percolate=-1&percolate_rt=0))

**170 - 182 516.5998 1546.7777 1546.8508 -0.0730 0 R.VPLQPPYQLHEVK.I**  ([Ions score 59](http://www.matrixscience.com/cgi/peptide_view.pl?file=../data/20100928/FteTlzESe.dat&query=36&hit=1&index=PRS28_MOUSE&px=1&section=5&ave_thresh=31&_ignoreionsscorebelow=0&report=0&_sigthreshold=0.05&_msresflags=1089&_msresflags2=2&percolate=-1&percolate_rt=0))

**170 - 182 516.9517 1547.8332 1546.8508 0.9824 0 R.VPLQPPYQLHEVK.I**  ([Ions score 22](http://www.matrixscience.com/cgi/peptide_view.pl?file=../data/20100928/FteTlzESe.dat&query=37&hit=1&index=PRS28_MOUSE&px=1&section=5&ave_thresh=31&_ignoreionsscorebelow=0&report=0&_sigthreshold=0.05&_msresflags=1089&_msresflags2=2&percolate=-1&percolate_rt=0))

**183 - 189 414.2228 826.4311 826.4548 -0.0237 0 K.IPIQDNK.S**  ([Ions score 44](http://www.matrixscience.com/cgi/peptide_view.pl?file=../data/20100928/FteTlzESe.dat&query=1&hit=1&index=PRS28_MOUSE&px=1&section=5&ave_thresh=31&_ignoreionsscorebelow=0&report=0&_sigthreshold=0.05&_msresflags=1089&_msresflags2=2&percolate=-1&percolate_rt=0))

1. Bottom of Form

**SUPPLEMENTARY REFERENCES**

1. Karlson U, Pejler G, Tomasini-Johansson B & Hellman L (2003) Extended substrate specificity of rat mast cell protease 5, a rodent alpha-chymase with elastase-like primary specificity. J. Biol. Chem. 278: 39625–39631.
2. Sharma N, Oikonomopoulou K, Ito K, Diamandis EP, Hollenberg MD & Rancourt DE (2008) Substrate specificity determination of mouse implantation serine proteinase and human kallikrein-related peptidase 6 by phage display. Biol. Chem. 389: 1097-1105
3. Karlson U, Pejler G, Froman G & Hellman L (2002) Rat mast cell protease 4 is a beta-chymase with unusually stringent substrate recognition profile. J. Biol. Chem. 277: 18579–18585.
4. Oikonomopoulou K, Hansen KK, Saifeddine M, Tea I, Blaber M, Blaber SI, Scarisbrick I, Andrade-Gordon P, Cottrell GS, Bunnett NW, Diamandis EP & Hollenberg MD (2006) Proteinase-activated receptors, targets for kallikrein signaling. J Biol. Chem. 281(43): 32095-112.
5. Al-Ani B & Hollenberg MD (2003) Selective tryptic cleavage at the tethered ligand site of the amino terminal domain of proteinase-activated receptor-2 in intact cells. J. Pharmacol. Exp. Ther. 304: 1120–1128.
6. Kawabata A, Saifeddine M, Al-Ani B, Leblond L & Hollenberg MD (1999) Evaluation of proteinase-activated receptor-1 (PAR1) agonists and antagonists using a cultured cell receptor desensitization assay: activation of PAR2 by PAR1-targeted ligands. J. Pharmacol. Exp. Ther. 288: 358–370.
7. Martin BR, Giepmans BN, Adams SR & Tsien RY (2005) Mammalian cell-based optimization of the biarsenical-binding tetracysteine motif for improved fluorescence and affinity. Nature Biotech. 23: 1308-1314.
8. Kao JP, Harootunian AT & Tsien RY (1989) Photochemically generated cytosolic calcium pulses and their detection by fluo-3. J. Biol. Chem. 264: 8179–8184.
9. Minta A, Kao JP & Tsien RY (1989) Fluorescent indicators for cytosolic calcium based on rhodamine and fluorescein chromophores. J. Biol. Chem. 264: 8171–8178.
10. Compton SJ, Cairns JA, Palmer KJ, Al-Ani B, Hollenberg MD & Walls AF (2000) A polymorphic protease-activated receptor 2 (PAR_2_) displaying reduced sensitivity to trypsin and differential responses to PAR agonists. J. Biol. Chem. 275: 39207–39212.
11. Dulon S, Cande C, Bunnett NW, Hollenberg MD, Chignard M & Pidard D (2003) Proteinase-activated receptor-2 and human lung epithelial cells: disarming by neutrophil serine proteinases. Am. J. Respir. Cell Mol. Biol. 28: 339–346.
12. Dulon S, Leduc D, Cottrell GS, D'Alayer J, Hansen KK, Bunnett NW, Hollenberg MD, Pidard D & Chignard M (2005) Pseudomonas aeruginosa elastase disables proteinase-activated receptor 2 in respiratory epithelial cells. Am. J. Respir. Cell Mol. Biol. 32: 411–419.
