# Supplementary material for: Implantation Serine Proteinase 1 Exhibits Mixed Substrate Specificity that Silences Signaling via Proteinase-Activated Receptors
Source: PLoS One. 2011 Nov 23;6(11):e27888. doi: 10.1371/journal.pone.0027888 (PMC3223204; doi:10.1371/journal.pone.0027888)
Supplement: Figure S1 — Expression of ISP1. (A) Fermentation parameters including pH, temperature, % dissolved oxygen (DO) and packed cell volume (PCV) recorded during a typical fermentation run (10 litre working volume), (B) Feeding rates of glycerol, methanol and oxygen shown in % figures during a typical fermentation run (10 litre working volume), (C) Expression of recombinant ISP1 as determined by Western blot analysis using monoclonal anti-mouse ISP1 antibodies (samples were withdrawn at different time intervals), lane 1–30 hrs., lane 2–40 hrs., lane 3–50 hrs., lane 4–60 hrs., lane 5–70 hrs., lane 6–80 hrs., lane 7–90 hrs., lane 8–100 hrs. (DOC) [file pone.0027888.s002.doc]

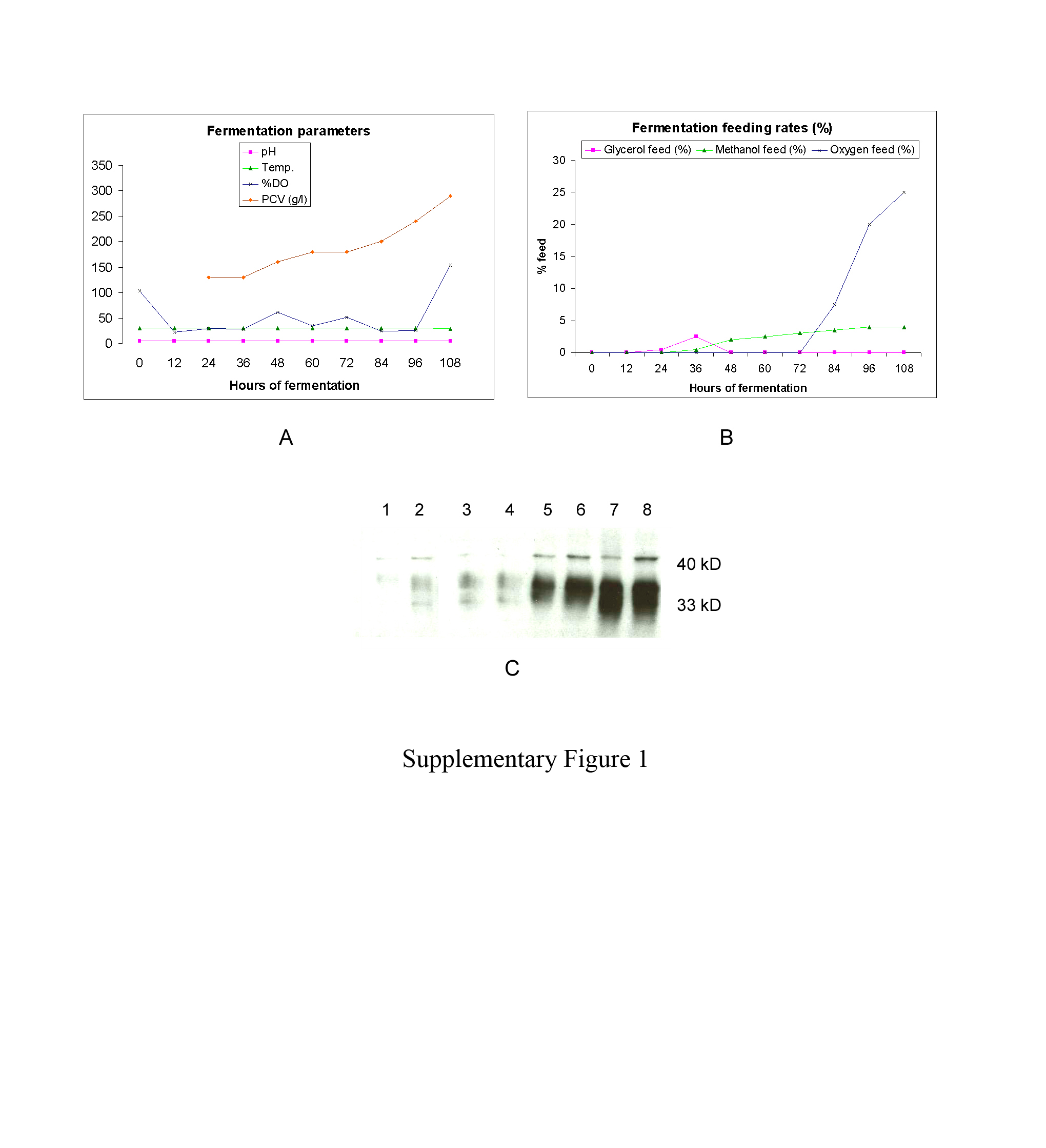


Expression of recombinant ISP1; (A) Fermentation parameters including pH, temperature, % dissolved oxygen (DO) and packed cell volume (PCV) recorded during a typical fermentation run (10 litre working volume), (B) Feeding rates of glycerol, methanol and oxygen shown in % figures during a typical fermentation run (10 litre working volume), (C) Expression of recombinant ISP1 as determined by Western blot analysis using monoclonal anti-mouse ISP1 antibodies (samples were withdrawn at different time intervals), lane 1 - 30 hrs., lane 2 - 40 hrs., lane 3 – 50 hrs., lane 4 – 60 hrs., lane 5 – 70 hrs., lane 6 – 80 hrs., lane 7 – 90 hrs., lane 8 – 100 hrs.
