# Supplementary material for: Implantation Serine Proteinase 1 Exhibits Mixed Substrate Specificity that Silences Signaling via Proteinase-Activated Receptors
Source: PLoS One. 2011 Nov 23;6(11):e27888. doi: 10.1371/journal.pone.0027888 (PMC3223204; doi:10.1371/journal.pone.0027888)
Supplement: Figure S2 — ES-MS analysis of reaction mixture showing the detection of FYIQ as a cleavage product of RRFYIQ when incubated with ISP1. (DOC) [file pone.0027888.s003.doc]

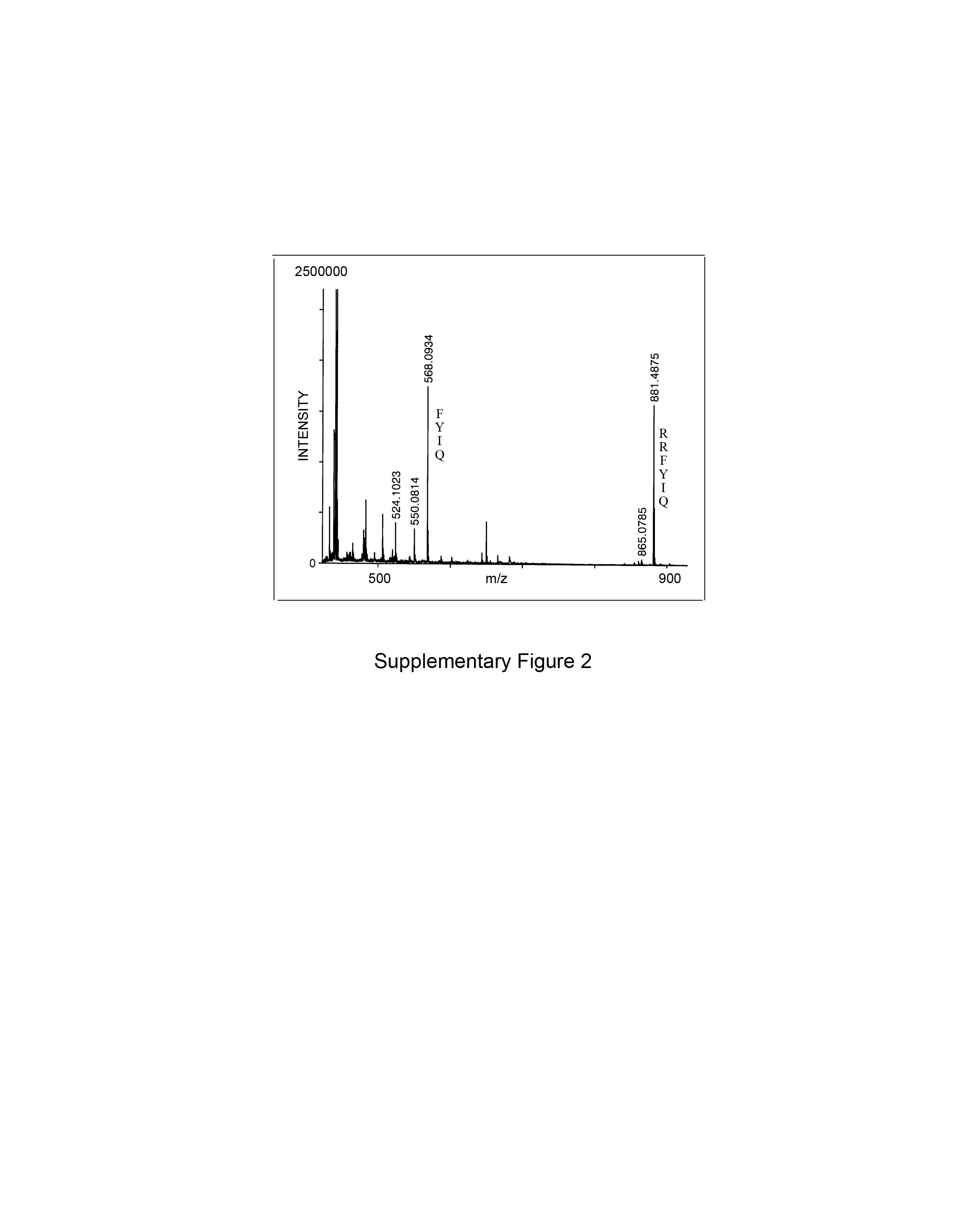


**Supplementary Figure 2:** ES-MS analysis of reaction mixture showing the detection of FYIQ as a cleavage product of RRFYIQ when incubated with ISP1.
