# Supplementary material for: Implantation Serine Proteinase 1 Exhibits Mixed Substrate Specificity that Silences Signaling via Proteinase-Activated Receptors
Source: PLoS One. 2011 Nov 23;6(11):e27888. doi: 10.1371/journal.pone.0027888 (PMC3223204; doi:10.1371/journal.pone.0027888)
Supplement: Figure S3 — ERK activation assay with rat PAR2 and control (pcDNA) transfected KNRK cells. No activation of ERK is observed upon incubation of cells with ISP1for 10 min. (DOC) [file pone.0027888.s004.doc]

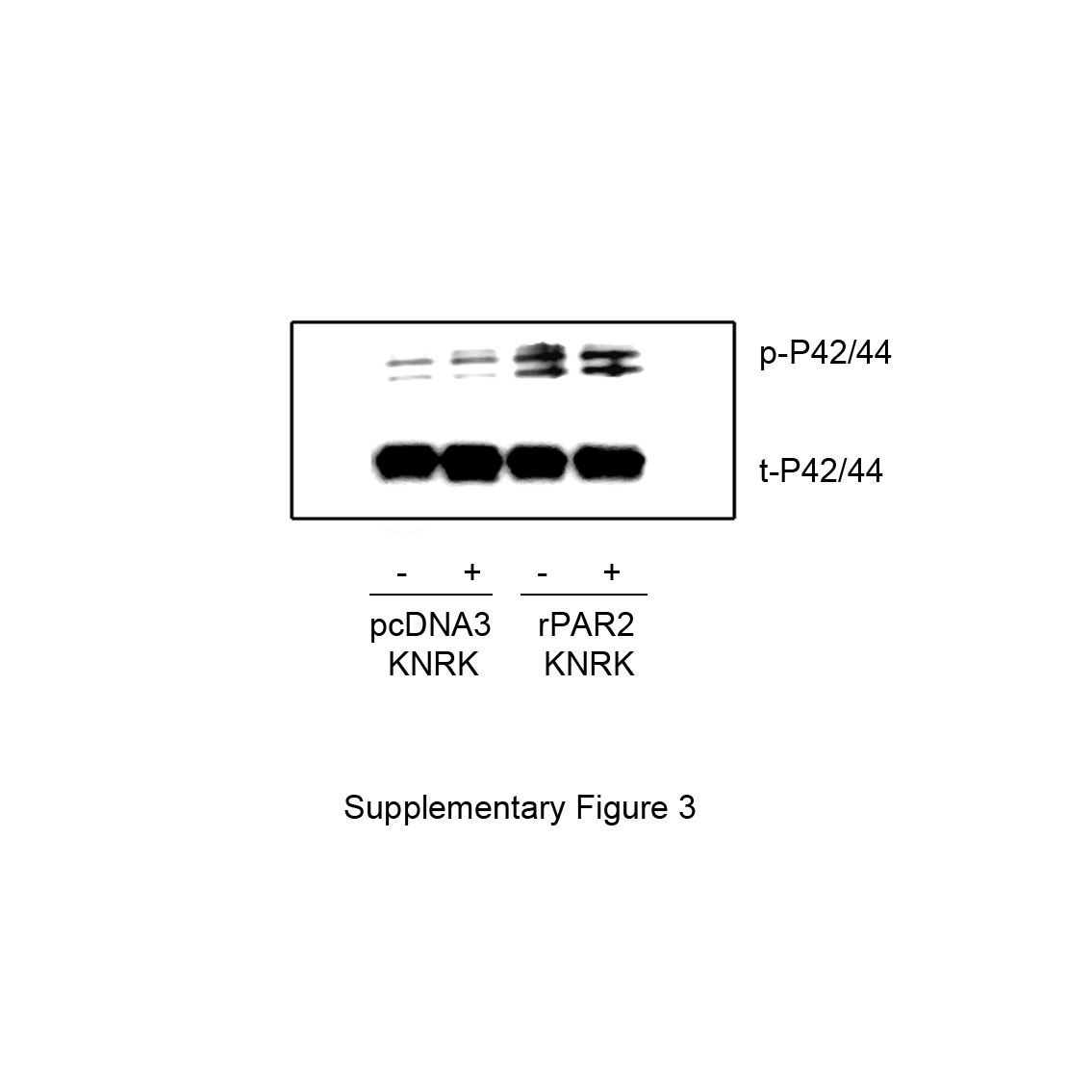


**Supplementary Figure 3:** ERK activation assay with rat PAR_2_ and control (pcDNA) transfected KNRK cells. No activation of ERK is observed upon incubation of cells with ISP1for 10 min.
